# Exploiting endocytosis for transfection of mRNA for cytoplasmatic delivery using cationic gold nanoparticles

**DOI:** 10.3389/fimmu.2023.1128582

**Published:** 2023-05-09

**Authors:** Muriel F. Gustà, Michael J. Edel, Vivian A. Salazar, Belén Alvarez-Palomo, Manel Juan, Massimo Broggini, Giovanna Damia, Paolo Bigini, Alessandro Corbelli, Fabio Fiordaliso, Alexander Barbul, Rafi Korenstein, Neus G. Bastús, Víctor Puntes

**Affiliations:** ^1^ Institut Català de Nanociència i Nanotecnologia (ICN2), Consejo Superior de Investigaciones Científicas (CSIC), The Barcelona Institute of Science and Technology (BIST), Barcelona, Spain; ^2^ Vall d’Hebron Institut de Recerca (VHIR), Barcelona, Spain; ^3^ Biomedical Research Networking Center in Bioengineering, Biomaterials and Nanomedicine (CIBER-BBN), Madrid, Spain; ^4^ Hospital Clínic de Barcelona, Servei Immunologia-IDIBAPS, Barcelona, Spain; ^5^ Unit of Anatomy and Embryology, Universitat Autònoma de Barcelona, Faculty of Medicine, Barcelona, Spain; ^6^ University of Western Australia, Faculty of Medicine, Discipline of Medical Sciences and Genetics, School of Biomedical Sciences, Perth, WA, Australia; ^7^ Banc de Sang i Teixits, Cell Therapy Service, Barcelona, Spain; ^8^ IRCCS‐Istituto di Ricerche Farmacologiche Mario Negri, Milano, Italy; ^9^ Tel Aviv University, Sackler School of Medicine, Tel Aviv-Yafo, Israel; ^10^ Institució Catalana de Recerca i Estudis Avançats (ICREA), Barcelona, Spain

**Keywords:** gold nanoparticles, cationic, transfection, gene therapeutics, safety

## Abstract

**Introduction:**

Gene therapy holds promise to cure various diseases at the fundamental level. For that, efficient carriers are needed for successful gene delivery. Synthetic ‘non-viral’ vectors, as cationic polymers, are quickly gaining popularity as efficient vectors for transmitting genes. However, they suffer from high toxicity associated with the permeation and poration of the cell membrane. This toxic aspect can be eliminated by nanoconjugation. Still, results suggest that optimising the oligonucleotide complexation, ultimately determined by the size and charge of the nanovector, is not the only barrier to efficient gene delivery.

**Methods:**

We herein develop a comprehensive nanovector catalogue comprising different sizes of Au NPs functionalized with two different cationic molecules and further loaded with mRNA for its delivery inside the cell.

**Results and Discussion:**

Tested nanovectors showed safe and sustained transfection efficiencies over 7 days, where 50 nm Au NPs displayed the highest transfection rates. Remarkably, protein expression was increased when nanovector transfection was performed combined with chloroquine. Cytotoxicity and risk assessment demonstrated that nanovectors are safe, ascribed to lesser cellular damage due to their internalization and delivery via endocytosis. Obtained results may pave the way to design advanced and efficient gene therapies for safely transferring oligonucleotides.

## Introduction

Developing efficient gene therapies depends on the means for transferring oligonucleotides (DNA or RNA) into the cell. The most common vectors used are replication-defective vector systems based on two types of viruses: retroviruses and adenoviruses. These vectors have been engineered to drastically reduce the transcriptional activity of the virus, virtually eliminating the possibility of viral reactivation. However, uncontrolled integration into the genome can potentially lead to insertional mutagenesis if the integration of vector DNA into host cells is placed near an oncogene, posing serious concerns in their clinical application (1). Additionally, their production is complex, and their production under Good Manufacturing Practices is burdened with strict regulations. Last but not least, DNA-insertion therapies in the context of CAR-T cell therapy, where ex-vivo transfection reprograms T cells to destroy B cells in the case of leukaemia, deplete patients of B cells for life with the corresponding health and societal burden. As an alternative, the idea of mRNA transfection will lead to transient cell therapy, dramatically reducing genetic therapy side effects. In addition, the transfection with mRNA has to be performed in the cytoplasm, opening the venue for non-viral transfection vectors.

Unlike viral vectors, nonviral ones rely on forming noncovalent assemblies between mRNA (a polyanion), cationic polymers, and lipidic moieties. Although a leading class of synthetic gene-delivery vehicles, cationic polymers suffer from high toxicity, and their efficiency does not compare to viral systems (2). To overcome these limitations, the delivery of oligonucleotides using nanovectors, a nanoparticle-based transfection vector, is attracting increasing attention (3–6). This includes lipidic and polymeric NPs composed of biocompatible units that self-assemble encapsulating nucleic acids. Also, inorganic NPs which can be functionalized with DNA or mRNA and can be used for *in vitro* transfection applications. Among the candidates, Au nanoparticles (NPs) are remarkably interesting due to their small size and monodispersity, low cytotoxicity, low immunogenicity, biocompatibility, straightforward synthesis and easy functionalization (7). Indeed, the chemical functionalization of Au NPs with cationic molecules (5, 8–12), especially polyethylenimine (PEI) molecules (13, 14), favours efficient oligonucleotide adsorption by high-affinity electrostatic interactions, ultimately enabling transfection. In any case, the interactions between the innate immune system and nanoparticles and derived objects can be especially immunogenic. Thus, purity, solubility, and surface state has to be precisely controlled (15).

The efficient loading of the nanovectors is ultimately determined by its size and charge. By adjusting its size, its loading capacity is modified (16). Thus, the load vs carrier ratio increases as the vector becomes smaller. Similarly, the loading depends on the charge of the nanovector. Cationic polymers possess many positive charges providing strong interactions with oligonucleotides, which are negatively charged. As simple as that, results suggest that optimising the oligonucleotide complexation is not the only barrier to gene delivery (8, 9, 13, 14, 17). A higher grafting density of PEI does not always result in more compact and smaller complexes with mRNA, intended to prevent degradation and facilitate cytosolic mobility. Besides, approaches that work for one cell line might not perform well for others, suggesting that the mechanisms that NPs use for cell entry and cell trafficking are essential factors to consider when designing efficient transfection nanovectors (18).

The second aspect is the cellular uptake of the nanovector. Endocytosis is the primary mechanism for the uptake of nanovector- oligonucleotide complexes (19). During this process, the loaded nanovector is engulfed by the cell membrane and delivered into the cell within a vesicle. The internalization process depends on nanovector size and charge. The highest cellular internalization is observed for NPs in the size ranges of 25–50 nm (20). Alternatives for cell entry other than endocytosis are membrane permeation and membrane fusion, which often result in high toxicity, especially permeation (21, 22). Similarly, surface charge determines cellular uptake. The negatively charged cell membrane enhances the uptake of positively charged NPs. However, the uptake of positively charged NPs may disrupt the integrity of the cell membrane and lead to an increase in toxicity (23) unless size and structure are provided.

The third aspect is the endosomal escape of the nanovector. Once inside the cell, the nanovector complex is trapped within a vesicle. Several mechanisms have been identified to escape the endosome into the cytosol, the most popularly known as the “proton sponge mechanism” (24). The proton sponge mechanism relies on the fact that present amines are not protonated under physiological conditions. These basic moieties can buffer the decreasing pH within the endosome. As more protons are pumped, more counter ions (mainly chloride) influx into the endosome for electroneutrality. This fact increases the osmotic pressure and, ultimately, the passive diffusion of water into the endosome. Consequently, the endosome continues to swell until the increasing membrane stress leads to membrane rupture and a release of the contents.

In this context, the study’s main objective is to design and develop transfection nanovectors for releasing nucleic acids as messenger RNA (mRNA) inside the cell ready for transfection. The introduction of mRNA by nonviral transfection vectors allows the gene’s transient expression, which presents several medical advantages. For instance, note that people treated with DNA CAR-T cell immunotherapy get deprived of B cells for life (25, 26). The nanovector consists of a cationic Au NP where mRNA is adsorbed for further delivery into the cytosol. There are two critical points in the development of the nanovector: i) the tight absorption of enough mRNA and ii) the efficient cytoplasmic release of the mRNA following the “proton sponge” effect. The number of mRNA molecules adsorbed onto the nanovector is expected to depend on the size of the nanovector and the density of amino groups present at their surface, and this density will also determine the efficiency of the cytoplasmic release. In addition, the absorption of the mRNA onto the NP, as occur with other molecules as proteins, protects them from degradation.

Thus, a nanovector catalogue has been developed comprising different sizes of Au NP cores, later functionalized with two different cationic molecules. These nanovectors were further loaded with mRNA for their application as nonviral transfection vectors. Cationic molecules have already been used as transfection agents, but with significant toxicity concerns (27–30). However, its absorption to the NP’s surface promotes the loss of flexibility and the membrane pore formation ability. Instead, PEI-derived NPs (nanovectors) attach to the cell membrane, not crossing it but inducing endocytosis. Consequently, its toxic aspect is eliminated thanks to nanoconjugation. Indeed, detoxification by nanoconjugation has been observed before in different systems (31). Thus, the monolayer coverage of Au NPs allows for tuning the charge and structure to maximize transfection efficiency while reducing associated toxicity.

## Results and discussion

### Synthesis, cationic functionalization and mRNA loading of citrate-stabilized Au NPs

The first critical point in the design of the nanovector is the efficient adsorption of mRNA for their further delivery into the cytosol. The number of molecules adsorbed onto the nanovector depends on the size and density of amino groups at their surface. Therefore, a nanovector catalogue comprising different sizes of Au NPs functionalized with two different cationic molecules and further loaded with mRNA was developed. By adjusting the size of the Au NPs and the nature of the cationic molecule, their loading capacity was modified. Characterization by UV-Vis spectroscopy, DLS and Z-Potential was performed at each step and summarized in **Table 1**. The sizes of choice were 5, 20 and 50 nm as this size regime favours NP’s endocytosis (32–35) and presents proven stability in physiological media (36, 37). As synthesized Au NPs were further functionalized with either 11-amino-1-undecanethiol acid (AUT) or polyethyleneimine 2kDa (PEI). Both cationic molecules contain amine terminal groups providing a positive charge to the Au NPs at physiological pH while differing in their molecular structure and density of amino groups. While AUT consists of an 11-carbon chain with a terminal amine and a thiol group in the opposite site that pseudo-covalently binds to gold. The employed PEI is a branched polymer that binds electrostatically to the NP. Linear PEI was also tested but increased toxicity without increasing efficacy (data not shown).

**Table 1:** Characterization of the nanovector catalogue.

	NPs	NPs-AUT	NPs-AUT+RNA	NPs-PEI	NPs-PEI+RNA
Au 5 nm					
SPR (nm)	510	521	533	516	519
DLS (nm)	6.1 ± 1.2	11.6 ± 3.6	62.7 ± 25.5	15.6 ± 4.9	15.1 ± 3.6
Z-Pot (mV)	-59.7	+5.8	-10.3	+26.2	+9.3
Au 20 nm					
SPR (nm)	522	526	531	523	524
DLS (nm)	30.4 ± 10.6	37.0 ± 13.3	54.6 ± 22.9	38.9 ± 15.3	44.9 ± 19.6
Z-Pot (mV)	-44.4	+18.6	-28.6	+34.4	+21.7
Au 50 nm					
SPR (nm)	531	533	540	531	534
DLS (nm)	49.6 ± 14.5	58.8 ± 25.9	75.0 ± 35.7	67.5 ± 30.2	72.8 ± 26.4
Z-Pot (mV)	-41.2	+31.2	-25.7	+44.3	+34.9

Summary of sizes, optical properties and surface charge of citrate-capped Au NPs of 5, 20 and 50 nm, cationic functionalized Au NPs with AUT of PEI, and loaded with mRNA. Note that the pH at which ζ-Potential of the citrate-capped NPs was measured (pH~8.6) is different from the other measurements, where NPs were dispersed in MES buffer (pH~5). This fact fundamentally impacts the surface charge of the NP.

**Figure 1:**
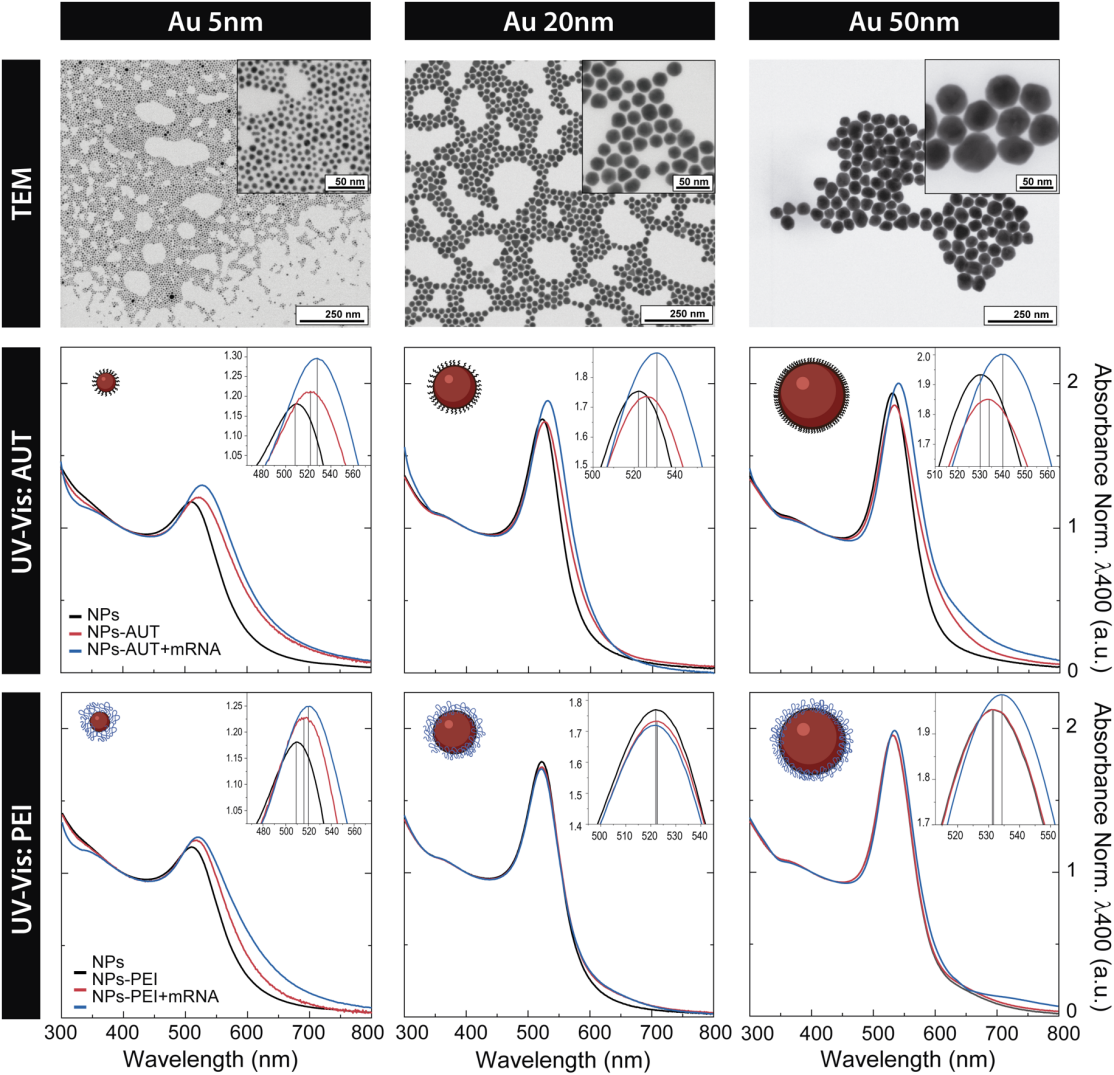
Nanovector Catalogue. Representative transmission electron microscopy images of highly monodisperse citrate-stabilized Au NPs of ∼5 nm, ∼20 nm, and ∼50 nm. UV-Vis spectra of citrate-capped Au NPs of 5, 20 and 50 nm, cationic functionalized Au NPs with AUT of PEI, and loaded with mRNA. Absorbance is normalized to λ400 nm for a better comparison.

Citrate-stabilized Au NPs were produced using a well-established seeded growth approach based on the citrate reduction of HAuCl_4_ (38, 39). Citrate is a good capping agent because it only binds loosely at the Au NP surface, being easily replaced by other ligands such as thiol- or amine-containing ligands, which have higher binding affinities for Au surfaces (~ 45 Kcal/mol (40) and ~ 6 Kcal/mol (41), respectively). Representative transmission electron microscopy (TEM) images of 5 nm, 20 nm and 50 nm Au NPs are shown in **Figure 1**, revealing their high monodispersity and quasi-spherical morphology. Citrate-stabilized Au NPs are stable in aqueous media and display a well-defined surface plasmon resonance (SPR) peak that red-shifts and increases in intensity as NPs increases in size.

As synthesized citrate-stabilized Au NPs were functionalized with AUT and PEI by incubating them with an excess of the cationic molecule at pH 2.5 for 24 hours. Remarkably, under these extremely acidic conditions, the stability of citrate-capped Au NPs is compromised due to loss of electrostatic repulsion, and aggregation is only prevented by the effective conjugation of the selected molecules. Citrate has three pKa points which are 6.3, 4.7, and 3.1. Therefore, below pH~ 3, all the hydroxyl residues become protonated, losing their negative charge, and, in turn, NPs lose the electrostatic repulsion provided by the citrate capping. The presence of a self-assembled AUT monolayer provides colloidal stability to NPs through the positively charged amine-terminal residue that avoids aggregation by electrostatic repulsion. Therefore, appropriate conjugation conditions are critical to retaining NP stability during the coating process (see **Figure S1**, **Figure S2**).

Characterization by UV-Vis spectroscopy indicates the effective conjugation of Au NPs to cationic molecules. The absorption spectrum is sensitive to the Au NP environment, and an observable red-shift of the SPR band of about 6-10 nm can be seen once the Au NPs are functionalized with the cationic molecules (42, 43). The shift occurs within a few minutes and then remains unaltered, suggesting that the conjugation process is quick and that NPs are stable for long periods. The extent of the redshift depends on the Au NPs size, the structure of the cationic molecule and the nature of the anchoring group (42). Thus, smaller Au NPs functionalized with AUT molecules bond *via* thiol groups exhibit the largest SPR shift. Although PEI is relatively big, it interacts electrostatically with NP’s surface by the positively charged amine residues, not forming covalent bonds, ultimately leading to smaller SPR shifts.

The functionalization process was further assessed by dynamic light scattering (DLS). An apparent increase in the hydrodynamic size of the NPs is observed after conjugation. Remarkably, monomodal distribution profiles were obtained in all cases, indicating that cationic Au NPs are colloidally stable. Measurement of NP surface charge, performed by ζ-potential measurements, revealed that cationic functionalization leads to highly positively charged Au NPs. Independently of their size, an increased surface charge is observed for PEI than AUT conjugates, explained by the higher amine density of the PEI coating due to its branched polymeric nature (44).

Functionalized NPs were purified and redispersed in MES buffer (pH ~ 5.5) for their later loading with oligonucleotides. Based on our previous expertise (36, 37, 45), we added the cationic NPs to the oligonucleotide solution to maximize NPs surface coverage while avoiding uncontrolled aggregation during the mixture of both solutions (46). The ratio of oligonucleotide molecules to NP was optimized for each NP’s size tested to achieve the maximum loading while avoiding NP aggregation (**see** **Figure S3**). The incubation was performed at 4 °C for 24 hours to minimize the risk of nucleic acid degradation while ensuring surface saturation (**Figure S4** shows complete kinetics of the loading process). Samples were purified before characterization analysis.

As seen in **Figure 1**, at any given NP size and cationic coating, a red shift in the SPR peak position of about 1-2 nm can be observed, confirming the effective oligonucleotide loading. Accordingly, their hydrodynamic diameter increased, and their ζ-potential dropped due to the presence of the negatively charged mRNA molecules at the acidic pH of work (47). Yet, the increase in the hydrodynamic diameter is larger for PEI than for AUT-functionalized Au NPs. These results, coupled with the fact that the surface charge doesn’t invert to negative values in the case of PEI-functionalized Au NPs, point out that significant differences attributed to the disposition of the cationic coating at the NP’s surface could exist. As previously mentioned, AUT forms a regular self-assembled monolayer, whereas PEI, with a branched polymeric nature and high molecular weight, is most likely in a mushroom configuration. The fact that PEI-functionalized Au NPs remain positive after oligonucleotide loading has a significant impact on the interaction of the nanovectors with the cell surface and has a critical role in their transfection efficiency. Finally, aiming to calculate the loading of oligonucleotides to cationic Au NPs, quantification was performed by Nanodrop. For this, after the loading process, Au NPs were purified, and the supernatant was analyzed. Spectrophotometric results reveal a loading of 48% (9.3 ng/µl) for NPs-AUT and 45% (8.7 ng/µl) for NPs-PEI for 50 nm Au NPs. All in all, the presented results, along with the nanovector stability (**Figure S5**) and release studies (**Figure S6**), provided a deep understanding of the nanovectors properties and proved the high stability of the nanovector-oligonucleotide complex.

### Nanovector cytotoxicity studies

**Figure 2:**
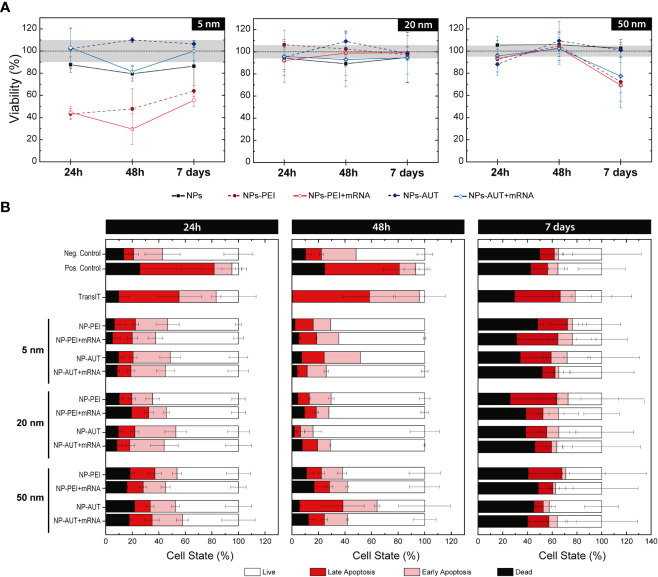
Nanovector Cytotoxicity Studies. The action of Au NP coated with PEI and AUT, and loaded with oligonucleotides was tested at different concentrations. **(A)** The percentage of resazurin reduction is shown at the maximum Au NP’s concentration. The dashed-black line corresponds to control values for reference, and the grey area overlaid to its relative standard deviation. **(B)** After 24h, 48h, or 7 days of HEK293 cells exposition to cationic NPs, alone or loaded with mRNA, Annexin V/PI staining was performed to analyze cell viability. Cell state was classified as live, in early or late apoptosis, or dead according to the relative intensity of each marker.

A key point in the success of nanovectors as nonviral transfection vehicles must be their low cytotoxicity. Other standard transfection methods, such as TransIT^®^ or Lipofectamine^®^, show good transfection rates but have very low cell viability. It is claimed that their internalization pathway is through membrane disruption, which ultimately causes their toxicity (48). In contrast, nanovectors internalize *via* endocytosis, significantly reducing the associated toxicity. To evaluate the safety of nanovectors in HEK293 cells, we first measured the cell viability by Resazurin reduction using Prestoblue and then Annexin V/PI staining was performed to analyze cell viability. (**Figure 2**).

Some previous studies reported that Au NPs cytotoxicity is mediated by their size, which inhibited the proliferation and triggered cell cycle arrest (49–51). However, the present results suggest no toxic effect related to NP size, at last in the studied range. Yet, cationic functionalization of the Au NPs did compromise cell viability and membrane integrity in some cases. A slight decrease in cell viability in HEK293 cells was determined with an exposition of 5 nm Au NPs coated with PEI and PEI-RNA at the maximum concentration of 3.3x10 (13) NP/mL. On the contrary, AUT-coated 5nm Au NP do not show any cytotoxic effect. Regarding 20 nm and 50 nm Au NPs, non-significant variation in the cell viability between Au-treated and non-treated cells was quantified even when exposing the cells to the highest concentration, corresponding to 2.7x10^12^ NP/mL and 3x10^11^ NP/mL, respectively.

To gain further insight into transfection-associated toxicity, HEK293 cells were exposed to nanovectors and sorted based on their viability state. Annexin V/PI staining was used to categorize cell populations as live, in early or late apoptosis, and dead. Viability controls were also included, cells without any treatment for the negative control and lethal H_2_O_2_ doses for positive cell death control. In this experiment, TransIT^®^ was also included to compare its toxicity with the NP-based nanovectors carrying similar amounts of oligonucleotides. Results show an evident difference in the viability profile of TransIT^®^ compared to nanovectors at short times. Cationic NPs, either loaded with mRNA or not, present a cell population distribution similar to the negative control. However, for TransIT^®^ exposed cells, the proportion of cells in a pro-apoptotic state is much higher, comparable with those treated with H_2_O_2_. The percentage of early and late apoptosis was maintained at 48 h. Accordingly, the dead cell proportion increased at each incubation time. In contrast, cells transfected with the nanovectors preserved their viability, and the apoptosis entrance was delayed. At 7 days post-exposure, viability values stabilized. Only a non-significant slight increase in the pro-apoptotic populations can be observed for TransIT^®^ and 5 nm Au NPs-PEI. These results confirm that NP-based formulations are safe for their use as delivery vectors. Cationic AuNPs non-loaded with DNA rapidly absorb negatively charged proteins from the medium losing their cationic charge and may cause uncontrolled aggregation (**Figure S5**, **Table S1**).

### Endosomal escape

**Figure 3:**
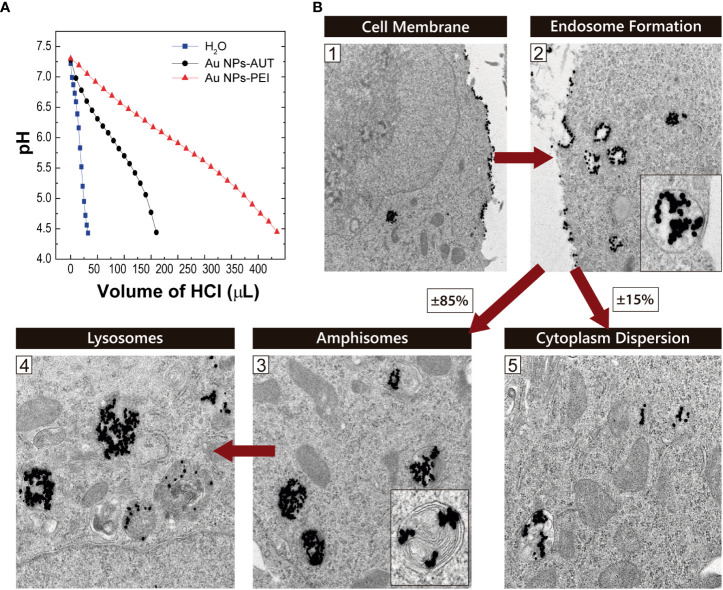
Endosomal Escape. **(A)** The proton buffer capacity of the cationic-coated Au NPs was analyzed by measuring the decrease in pH as a function of HCl volume added to the solution. **(B)** Au NP-PEI Intracellular Trafficking on HEK293 cells. Representative transmission electron microscopy images from HEK293 cells exposed to PEI-coated 20 nm Au NPs. The proposed internalization pathway is schematically represented at the different stages: (1) membrane attachment, (2) internalization in endosomes, maturation to (3) amphisomes and later (4) lysosomes. (5) Shows NPs dispersed in the cytoplasmic space after escaping from the endosomes. Red arrows indicate time evolution.

Reaching the cytoplasmic space is critical for the delivery of mRNA into the cytosol, where the ribosomes that will translate it into the coded protein are located. NPs with sizes ranging 5-100 nm enter the intracellular space *via* endocytosis, but the subcellular fate of the NPs will depend on their properties (32, 52). Upon vesicle formation, they enter into the endocytic pathway, where pH progressively decreases with the vesicle maturation process for digestion. However, NPs with pH buffering capacity can inhibit this process, disrupt the endosomal membrane, and escape from the endosome by the so-called proton sponge effect (53–55). Two parallel studies were performed to investigate the ability of the nanovectors to escape from the endosome (**Figure 3**).

First, the proton buffering capacity was tested *in vitro* by monitoring a solution’s acidification in cationic Au NPs. The proton sponge efficiency of the NPs was evaluated as a function of the HCl added; thus, H^+^ needed to reach the same pH value. As observed, PEI and AUT-coated NP solutions present a delay in the pH drop compared to the aqueous solution used as a reference. In detail, the Au NPs-PEI show a much greater ability to capture protons than NPs-AUT. This correlates with the amine concentration of the PEI-coated NPs being higher. These results were correlated with the findings from the NP intracellular trafficking study. The observation of cells exposed to cationic NP by TEM reveals that NPs follow an internalization pathway *via* endocytosis. After NPs-PEI stuck to the cell membrane, they were internalized in endocytic vesicles. In a typical endocytosis process, the maturation of these vesicles has several steps, from early and late endosomes to amphisomes, to finally reach the active lysosome stage, where the proteases digest the cargo. In this case, it could be observed that close to 85% of the internalized NPs-PEI were found either in amphisomes or in lysosomes. However, the 15% remaining were located dispersed in the cytoplasmic space. This fact entails that NPs-PEI escaped from the endocytic pathway at some point.

As discussed before, the coating of Au NPs with PEI confers proton sponge capacities allowing for an endosomal escape, as results suggest. However, the loading of cationic NP with oligonucleotides and their interfacing with biological media may impact on their interation with cells. In fact, the interaction with components from biological fluids -as the potential formation of a protein corona- could influence nanovectors cell internalization, distribution and fate (56). In our case, cells are transfected in serum free medium avoiding interaction with proteins in the cell culture.

### Transfection: GFP expression

The effectiveness of Au transfection in HEK293 cells was assayed using 5, 20 and 50 nm Au NP coated with PEI and AUT, loaded with green fluorescence protein, *GFP* mRNA. The GFP expression was analyzed at short (24 and 48 h) and long (7 days) times post-transfection, by flow cytometry and fluorescence microscopy. The observation of the transfected cells by fluorescence microscopy allowed for a first visual inspection of the samples to evaluate cell morphology and GFP expression qualitatively. In addition, the transfection efficiency from the total cell population was measured by flow cytometry.

**Figure 4:**
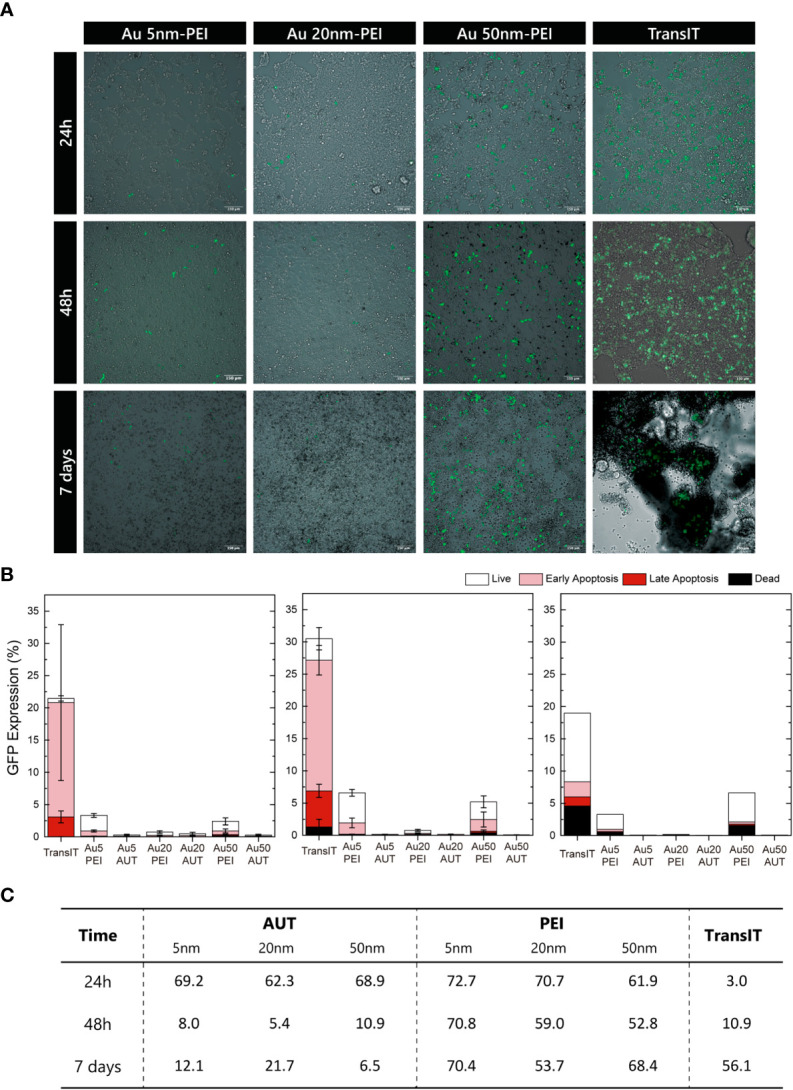
GFP Expression. **(A)** Wide-Field fluorescence overlaid onto Bright-Field images of transfected HEK293 cells at 24h, 48h, and 7 days. Scale bar = 150 µm. **(B)** Quantifying the nanovectors transfection based on the mean GFP expression at 24h, 48h, and 7 days. Cell viability was analyzed by Annexin V/PI iodine staining, and the cell state was classified as live, in early or late apoptosis, or dead. **(C)** Summary of the GFP-expressing live cells relative to the total GFP-expressing cells.

**Figures 4A, B** shows the transient expression of GFP. While all the Au NP-PEI could transfect the HEK293 cells, the transfection performed with the AUT-coated nanovectors was unsuccessful since no GFP expression was observed because the NPs did not reach the cytosol, either because they did not release the mRNA. Interestingly, the expression of the GFP protein was preserved for 7 days which could be adscribed to a slow mRNA from the nanovector (**Figure S6**). Some morphological and adherence changes were observed, especially after exposition to 5nm NPs-PEI and the commercial reagent TransIT^®^, consistent with necrosis as reported in the cytotoxicity studies. Additionally, to evaluate ribosomal occupancy and saturation different concentrations of mRNA were tested, which allowed to standardize the transfection protocol with nanovectors (**Figure S7**). It was observed that the transfected population increased with the amount of mRNA indicating that despite the high amounts of mRNA, the ribosomes were far from saturation, probably due to the slow and sustained release of mRNA in the cytosol.

Cellular viability was evaluated by flow cytometry using Annexin/PI combined staining to study apoptosis phenomena in those expressing GFP cells to gain further insights into the transfection events. Thus, GFP-positive cells were sorted into live, early or late apoptosis, or dead depending on the relative intensity of both viability markers. **Figure 4B** shows the expression in live cells at 24h, 48h and 7 days. HEK293 cells transfected with TransIT^®^ show the highest GFP fluorescence signal but with relatively high-intensity variability within the same population. Regarding the PEI-nanovectors, 50 nm NP show the highest efficiency on all time points, and GFP expressions are maintained for 7 days, while that from TransIT^®^ has started decreasing.

Interestingly, when GFP expression is reviewed as the proportion of live to total transfected cells (**Figure 4C**), differences between TransIT^®^ and nanovectors dramatically change. These results reveal a high and prolonged transfection efficiency of HEK293 cells by PEI-nanovectors, coupled with low cytotoxicity, since the apoptosis cell ratio is not enhanced. It can be seen that the amount of healthy transfected cells is similar in both cases, TransIT^®^ and AuNPs, but while there is a large population of transfected apoptotic cells with the former, they are almost none in the latter, especially at short times. Additionally, the observation of the samples under the CLSM enabled the image of the Au NPs simultaneously by reflectance mode (57–59) (**Figure S8**). These findings, coupled with the TEM observations of the NPs intracellular trafficking, may indicate that despite most of the cells having internalized nanovectors, they are not yet dispersed in the cytoplasmic space. Therefore, endosomal escape may happen later for these nanovectors, delaying the mRNA delivery and expression, which also agrees with the sustained GFP expression observed by flow cytometry. Additionally, variability in GFP expression could be ascribed to differences in the cell cycle stage between the HEK293 population (60).

Aiming to test the versatility of the developed nanovectors, different cells lines were also trasnfected with GFP mRNA loaded on 5, 20 and 50 nm Au NP-PEI. The transfection efficiency of the nanovectors on Jurkat cells, an immortalized line of human T lymphocyte cells that are used to study acute T cell leukaemia and RAW264.7 as a model of macrophage has been tested. Immune cells, and particularly T cells morphology is specially challenging for cytoplasmatic delivery since the majority of the cell volume is occupied by the cell nucleous. Obtained results demonstrate the capacity of the nanovectors to deliver mRNA to different cell lines (**Figure S9**).

### Transfection: Cloroqiuine Effect

**Figure 5:**
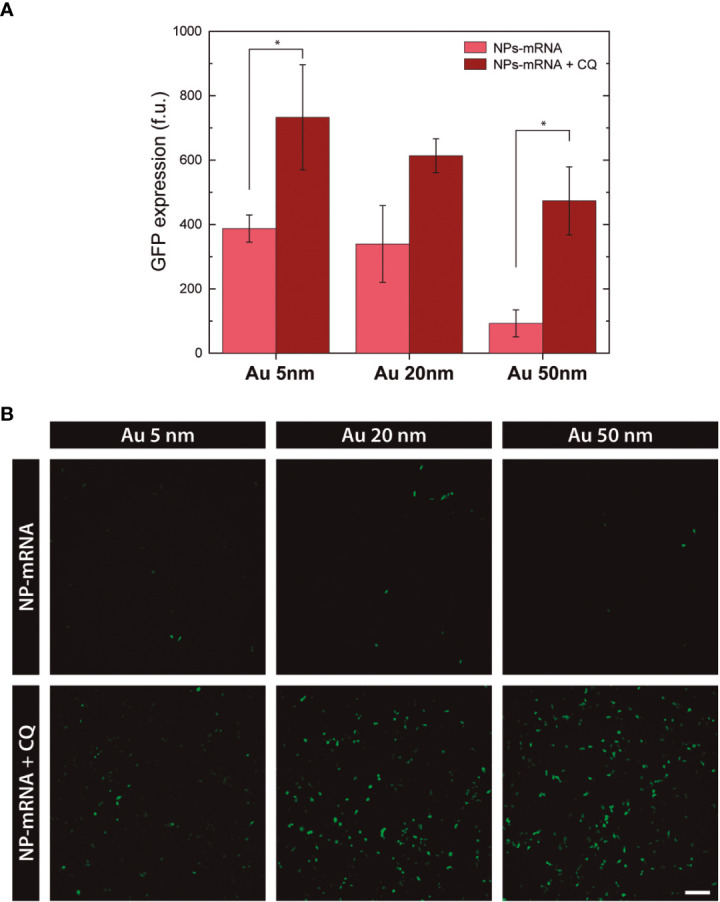
Effect of Chloroquine in transfection. **(A)** Transfection efficiency based on the mean GFP expression at 24h. (* p>0.05) **(B)** Wide-field Fluorescence images of HEK293 cells transfected with PEI-coated nanovectors, alone or in combination with chloroquine, at 24h. Scale bar = 150 µm.

Here, the combined use of nanovectors with chloroquine is explored to enhance the transfection efficiency (**Figure 5**). Chloroquine, as largely reported in the literature, is a common compound used to halt endosomal maturation, which in turn acts to boost NP endosomal escape by enhancing the proton sponge effect (61, 62). So, HEK293 cells were treated with chloroquine for 4 hours prior to transfection with PEI-nanovectors and GFP expression at 24h was visualized by wide-field fluorescence microscopy.

The statistical analysis from the GFP expression quantification shows a significant fluorescence signal increase for 5nm and 50nm PEI-nanovectors, and higher but not so significant for 20nm. Conversely, no enhancement effect was observed in cells transfected with AUT-coated nanovectors (data not shown). Accordingly, these results support the hypothesis that Au NPs-PEI reaches the cytoplasmic space *via* endosomal escape by the proton sponge mechanism. Further, this study provides new insights into the characterization of nanovectors that ensure suitable mRNA delivery into the cells without a cytotoxic effect.

## Conclusions

In this work role of cationic functionalized Au NPs as transfection vectors has been explored. Au NPs of 5 nm, 20 nm and 50 nm were synthesized with high monodispersity following a previously reported seeded-growth method. NPs were successfully coated with either AUT or PEI to provide cationic surface charge and then loaded with mRNA, which constitutes the nanovector construct. We studied stability in biological media and the ability to release the previously loaded mRNA over time. Nanovectors also displayed proton sponge capacity, and further microscopy studies suggested their delivery into the cytosol *via* endosomal escape. The use of PEI-coated Au NPs as mRNA delivery vectors showed a relatively low transfection efficiency but sustained GFP protein expression over 7 days, where 50 nm Au NPs displayed the highest transfection rates. Cytotoxicity and risk assessment performed by Prestoblue and Annexin V/PI assays demonstrated are safe and didn’t induce significant cell damage, except for 5 nm Au-PEI NPs that showed higher toxicity, similar to TransIT transfection control. Protein expression was increased when nanovector-mRNA transfection was performed combined with chloroquine. The mRNA transfection with NPs induced less cellular damage and mortality due to their internalization and delivery *via* endocytosis. All in all, cationic Au NPs were proven to be safe, nonviral vectors for mRNA delivery into cells, with a wide margin for improvement.

In conclusion, the introduction of mRNA into the cells for protein overexpression is an alternative to viral vectors that includes several advantages: no threat of mutagenic insertion, no threat of viral particle reactivation, accessible to dose control, and synthetic animal product-free production, which altogether makes it an attractive approach for clinical use since they allow for a transient expression of the desired gene. Not only that, but non-viral vectors have gained importance in recent years because of their safety in handling and ease of application compared with viral vectors. In contrast, they are customizable, non-pathogenic, relatively safe, and easily produced and scaled-up.

## Materials and methods

### Chemicals

Gold(III) chloride trihydrate (HAuCl_4_·3H_2_O), trisodium citrate (Na_3_C_6_H_5_O_7_), tannic acid (C_76_H_52_O_46_), potassium carbonate (K_2_CO_3_), amino-undecanethiol (AUT), poly-ethyleneimine branched Mn2000 (PEI), 2-(N-morpholino)ethanesulfonic acid buffer solution (MES), sodium hydroxide (NaOH), hydrogen chloride (HCl), oligonucleotide model 600-800 bases (D1626), Sodium Phosphate Dibasic (Na_2_HPO_4_), Sodium phosphate monobasic (NaH_2_PO_4_), poly-L-lysine, Paraformaldehyde (PFA), Triton-X, Bovine Serum Albumin (BSA), Sodium Chloride (NaCl), and Calcium Chloride (CaCl_2_) were purchased from Sigma-Aldrich. Dulbecco’s Modified Eagle Medium (DMEM), Foetal Bovine Serum (FBS), Hoechst 3342 (H1399), Prolong antifade mounting medium (11559306), Optimem Medium, Pacific Blue-Annexin V, Propidium iodide (PI), accutase, 4-(2-hydroxyethyl)-1-piperazineethanesulfonic acid buffer (HEPES), and Prestoblue were purchased from Thermo Fisher. Phalloidin Alexa Fluor 647 (ab176759) was purchased from Abcam. Clean CAP eGFP mRNA (5 moU) was purchased from Tebu-Bio. TransIT^®^-LT1 Transfection Reagent was purchased from MirusBio. All chemicals were used as received without further purification. Distilled water passed through a Millipore system (ρ = 18.2 MΩ) was used in all experiments. All glassware was first rinsed with acetone and then with Millipore water before use.

### Gold nanoparticle synthesis

5 nm citrate-stabilized Au NPs were producted following Piella et al. In detail, a 150 mL of freshly prepared reducing solution of sodium citrate (SC, 2.2 mM) containing 0.1 mL of tannic acid (TA, 2.5 mM) and 1 mL of potassium carbonate (K_2_CO_3_, 150 mM) was heated with a heating mantle in a 250 mL three-necked round-bottom flask under vigorous stirring. When the temperature reached 70°C, 1 mL of tetrachloroauric acid (HAuCl_4_, 25 mM) was injected. The colour of the solution changed rapidly to black-grey (less than 10 s) and then to orange-red in the following 1−2 min. The solution was kept at 70°C for 5 min more to ensure complete reaction of the gold precursor. Immediately after the synthesis and in the reaction same vessel, the sample was diluted by extracting 55 mL and adding 55 mL of SC (2.2 mM). When the temperature reached again 70°C, two injections of 0.5 mL of HAuCl_4_ (25 mM) on a time interval of 10 min were done. This growing step comprising sample dilution plus 2 injections of HAuCl_4_ was repeated until the particles reached the desired size.

20nm and 50nm citrate-stabilized Au NPs were produced following Bastus et al. In detail, a solution of 2.2 mM sodium citrate (SC) in Milli-Q water (150 mL) was heated with a heating mantle in a 250 mL three-necked round-bottomed flask for 15 min under vigorous stirring. A condenser was utilized to prevent the evaporation of the solvent. After boiling had commenced, 1 mL of HAuCl_4_ (25 mM) was injected. The colour of the solution changed from yellow to bluish grey and then to soft pink in 10 min. Immediately after the synthesis of the Au seeds and in the same reaction vessel, the reaction was cooled until the temperature of the solution reached 90°C. Then, 1 mL of a HAuCl_4_ solution (25 mM) was injected. After 30 min, the reaction was finished. This process was repeated twice. After that, the sample was diluted by extracting 55 mL of sample and adding 53 mL of Mili-Q water and 2 mL of 60 mM sodium citrate. This solution was then used as a seed solution, and the process was repeated again until the particles reached the desired size.

### Functionalization of nanoparticles

Functionalization of Gold Nanoparticles with AUT. First parameter explored for a stable functionalization of Au NPs was the concentration of AUT. For this, 20nm Au NPs were concentrated 10-fold relative to the synthesis concentration by centrifugation (conditions were set according the Stokes law for each particle size). Next, AUT solutions with concentrations ranging between 50-400 µM were prepared in HCl 10 mM (pH<3). NPs (10% to final volume) were rapidly added into the AUT solution under vigorous stirring. After 1h, samples were characterized by UV-Vis. Note that at pH values above 3, NPs aggregate and precipitate upon dispersion in the AUT solution. The positive charges of the amine residues of AUT interact with the negatively charged hydroxyl residues of citrate and crosslink triggering NPs aggregation. The conjugation time was analyzed by monitoring the NPs by UV-Vis from 5 min to 1 month. Finally, the purification process of the AUT-coated NPs was studied. The conjugated NPs were precipitated by centrifugation twice, and resuspended to the initial volume, first with HCl 2 mM and then with MES 10 mM.

Functionalization of Gold Nanoparticles with PEI. The optimal PEI concentration and pH were studied for Au NPs PEI-coating. On the first case, 50nm Au NPs were concentrated 10-fold relative to the synthesis concentration by centrifugation. Next, PEI solutions with concentrations ranging between 50-200 µM were prepared in HCl 34mM (pH~7). NPs (10% to final volume) were rapidly added into the PEI solution under vigorous stirring. After 1h, samples were characterized by UV-Vis. 10-fold concentrated 50nm Au NPs were conjugated to PEI (200 µM) at different pH conditions ranging from 2 to 7. NPs (10% to final volume) were rapidly added into the PEI solution under vigorous stirring. After 24h, samples were characterized by UV-Vis. The conjugated NPs were precipitated by centrifugation, resuspended to the initial volume with water and characterized again by UV-Vis.

### Loading of cationic gold nanoparticles with oligonucleotides

#### Optimization of the NP : RNA ratio

50 nm (at 3x10^11^) NP/mL) Au NPs coated with AUT were used, previously purified and dispersed in MES 10 mM. Single-stranded DNA (ssDNA) with a molecular weight similar to an average mRNA construct was used as an oligonucleotide model. For ssDNA loading, first 900 µl of 2-fold serial dilutions in MES 10mM were prepared, ranging from 53-0.41 µg/mL. Next, 100 µl of NPs were rapidly added onto the ssDNA and the mixture was gently homogenized. Thus, the final relative ssDNA : NP ratios ranged from [39-5000]. Samples were incubated for 24 h at 4°C under stirring. Next day, samples were characterized by UV-Vis spectroscopy, DLS and Z-Pot before and after purification. For purification, NPs were precipitated by centrifugation, supernatant was discarded and pellets were resuspended in MES 10 mM to the initial volume.

#### Loading kinetics

50 nm Au NPs coated with AUT were loaded with ssDNA. Briefly, 900µl of NPs dispersed in MES 10 mM were added onto 100 µl of ssDNA to a final ratio DNA : NP=300. Samples were kept at 4 °C under stirring. At each time point, 1 mL of sample was taken for characterization. For purification, NPs were precipitated by centrifugation, supernatant was stored for ssDNA quantification and pellets were resuspended in MES 10 mM to the initial volume. Conjugates were analyzed by UV-Vis spectroscopy, DLS and additionally Z-Potential was measured after purification. The quantification of the ssDNA loaded on the NPs was extrapolated from the measurement of the supernatants at 24h by Nanodrop (Nanodrop 2000 Spectrophotometer, ThermoFisher).

#### Stability of nanovectors

To study the stability of nanovectors, 50 nm Au NPs coated with AUT and PEI, alone or loaded with ssDNA, were used. For this, NP solution was diluted 1:10 in the media of study and incubated for 24h at 4 °C. Different biologically relevant media were tested: Optimem (pH 7.4) and Phosphate Buffer (PB) 10mM (pH 7.4). NPs dispersed in MES 10 mM (pH 5) were used as a control. NP stability was studied by UV-Vis and DLS. After 24 h samples were characterized. Au NPs were precipitated by centrifugation, the pellets were redispersed in the media of study and Z-Potential was measured.

### *In vitro* experiments

#### Cells culture

HEK293 cell culture was maintained in culture in 75 cm^2^ tissue culture flask using DMEM with heat- inactivated foetal bovine serum (FBS) at 10% at 37 °C and humidified 5% CO_2_.

#### TEM imaging of cultured cells exposed to NPs

HEK293 cells were seeded on a 10 cm petri dish at 100.000 cell/cm^2^ and incubated overnight. 20 nm Au NPs coated with PEI were added dropwise onto cell cultures and gently homogenized. At 24 h cells were fixed with 2.5% glutaraldehyde in 0.1 M PB. Next, samples were embedded in paraffin following a standard protocol. For observation, paraffin-embedded samples were sectioned using a ultra-microtome and transferred carbon-coated copper TEM grid.

#### Proton sponge efficiency of cationic gold nanoparticles

First, the pH of a cationic Au NPs solution was adjusted to 7.3 with NaOH. Then, pH was monitored continuously as a known volume of HCl (10 mM) was added dropwise on the Au NPs solution under stirring, until pH 4 was reached. A solution of Mili Q water was used as a control. The proton sponge efficiency of the cationic NPs was calculated based on the HCl volume added, normalized to Au surface (nm^2^).

### Cytotoxicity

#### Cytotoxicity assessment of nanovectors

The action of Au NPs in the viability of the HEK-293 cells was evaluated by PrestoBlue and Annexin V/Propidium Iodide assay, according to the manufacturer’s recommendations.

#### Prestoblue

HEK293 cells were seeded to 1x10^5^ cell/mL in 96-well plate during 24 hours before to Au NPs exposition. Serial dilutions of nanoparticles were added at final concentration ranging from 3.3x10^13^ - 1.2x10^11^ for Au 5nm NPs/mL, 2.7x10^12^) - 1.0x10^10^ for Au 20 nm NPs/mL and 3x10^11^ - 1.1x10^9^ for Au 50nm NPs/mL. To assay the ratio oligonucleotide:NPs cytotoxicity the PEI/AUT nanoparticles were loaded with oligonucleotide. After 24 h, 48 h and 7 days cell viability was measured. For this, 10 µL of PrestoBlue was added to each well, plates were incubated for 2h and fluorescence was measured (λ_ex_531nm, λ_em_572nm) by Varioskan LUX (Thermo Fisher Scientific). All experiments were carried out in triplicate, and data was treated and calculated with OriginLab software.

#### Annexin V/propidium iodide

To determinate the cell viability, HEK293 cells were stained with Pacific Blue- Annexin V/propidium iodide (PI) in accord with the manufacturer’s recommendations to determine cell viability. Briefly, HEK293 cells were collected by cell detachment using accutase and washed with PBS. After centrifugation cells were resuspended in 100 µL of Annexin binding buffer (10 mM HEPES, 140 mM NaCl and 2.5 mM CaCl_2_). 5 µL of Annexin V and PI (1 mg/mL) were added and incubated at room temperature for 15 minutes. After the incubation period, additional 400 µL of the binding buffer was added. Acquisition was configured to stop after recording 10,000 events within the HEK293 cell population.

### Transfection efficiency

#### Transfection of mRNA with nanovectors

To evaluate the transfection capacity of gold nanoparticles coated with PEI and AUT, HEK- 293 cells were cultured in DMEM with FBS 10% in 24-well plate at 50.000 cells/mL. The transfection was performed with 60-70% confluence and final mRNA concentration of 1000 ng. After 2h the incubation at 37°C the DMEM medium was removed and replaced for 900 µL of Optimem medium. Next, specific colloidal ratios [mRNA : NP] were added (100 µL) for each nanoparticle size for 5 nm Au NPs [5:1], 20 nm [50:1] and 50 nm [300:1]. The next day 100µL of FBS were added to each well and left for 48h and 7 days after transfection process. The transfection and cell viability percentages were evaluated by confocal microscopy and flow cytometry.

#### Chloroquine effect

To further inside to the proton sponge mechanism the transfected HEK-cells were treated with chloroquine at 20 μM for 4 hours before nanovector transfection, performed as described above. At 24h cells were visualized by Wide-Field fluorescence microscopy and GFP signal intensity quantified. Statistical analysis was performed by the 2-way ANOVA test, using the GraphPad Prism software. For significance, p>0.005 was considered.

#### Flow cytometry

The percentage of Green Fluorescence Protein (GFP) expression after transfection was analyzed with BD LSRFortessa™ Cell Analyzer. Forward and side-scatter areas (FSC-A, SSC-A) in a linear scale were used to gate HEK293 population, and GFP expression was detected by excitation through 480-500nm. To determinate the cell viability, HEK293 cells were stained with Pacific Blue- Annexin V/propidium iodide (PI) in accord with the manufacturer’s recommendations. Briefly, HEK293 cells were collected by cell detachment using accutase and washed with PBS. After centrifugation cells were resuspended in 100 µL of Annexin binding buffer (10mM HEPES, 140mM NaCl and 2.5mM CaCl_2_). 5 µL of Annexin V and PI (1mg/mL) were added and incubated at room temperature for 15 minutes. After the incubation period, additional 400 µL of the binding buffer was added. Acquisition was configured to stop after recording 10,000 events within the HEK293 cell population.

#### Wide-field fluorescence microscopy

Au nanoparticles transfection efficiency in HEK293 cells was calculated by GFP expression analyzed by Wide-Field microscopy. To this end, HEK293 cells were visualized in Thunder Wide-Field Fluorescence Microscope (Leica). For GFP imaging, a 475 nm LED was used for excitation while the emission channel was set to 506-532 nm. For deconvolution of each image, we utilized the algorithm Small Volume Computational Clearing (SVCC).

## Data availability statement

The raw data supporting the conclusions of this article will be made available by the authors, without undue reservation.

## Author contributions

MB, GD, PB, RK, ME, MJ, NB, VP conceived of the presented idea MG, ME, VS, AA-P, AB, AC, and FF designed and performed the experiments. All authors contributed to the article and approved the submitted version.
