# Supplementary material for: Exploiting endocytosis for transfection of mRNA for cytoplasmatic delivery using cationic gold nanoparticles
Source: Front Immunol. 2023 May 9;14:1128582. doi: 10.3389/fimmu.2023.1128582 (PMC10205015; doi:10.3389/fimmu.2023.1128582)
Supplement: Supplementary file 1 [file DataSheet_1.pdf]

SUPPLEMENTARY INFORMATION

Exploiting Endocytosis for Transfection of mRNA for Cytoplasmatic Delivery Using Cationic Gold Nanoparticles.

Au NPs Conjugation to AUT.

The effect of concentration, conjugation time, and the purification process was studied for AUT. Results are displayed in Figure S1. As observed, NPs were conjugated to increasing concentrations of AUT from 50 µM to 400 µM. The conjugation process was performed by incubating the NPs at 1.5×10^{12} NP/mL, with an excess of AUT from 3.5 to 30 times of molecules in solution – equivalent to the theoretical footprint area – at pH 2.5. The decrease in absorbance intensity at SPR and the concomitant increase within the range of 600-800 nm suggest that the stability of the conjugates is compromised at lower AUT concentrations tested, which translates into higher Aggregation Parameter (AP) values. As seen on the inset of Figure S1A, AP decreases with increasing AUT concentration. It shows a minimum value at 200 µM, where it reaches a plateau. Thus, 200 µM was set as a standard AUT concentration for Au NPs functionalization.

The kinetics of the conjugation process and the long-term stability of the sample were studied by analysing time-dependent measurements of absorbance spectra of AUT-functionalized NPs (NPs-AUT) (Figure S1B). Representative time points were chosen, 5 min, 5 days and 1 month. AUT conjugation appears quick since the shift occurred within a few minutes. Then, spectra remained unaltered, indicating that the solution was stable for weeks. These results agree with the high affinity of gold towards the SH residue present in AUT to form a pseudo-covalent thiol-bond on the NP's surface.

After the conjugation, a purification process is needed to eliminate all the non-conjugated AUT molecules in the solution, an essential step to avoid non-specific interactions or toxicity. For every purification step, NPs-AUT were precipitated by centrifugation and supernatants were discarded. A first wash is performed by resuspending the pellets with HCl 2 mM, to maintain the pH in acidic conditions (pH>3) and eliminating citrate traces that might cause NP crosslink and precipitation. Next, a second wash step is performed, resuspending the NPs-AUT in 10 mM 2-(N-morpholino)ethanesulfonic acid buffer (MES) (pH~5).

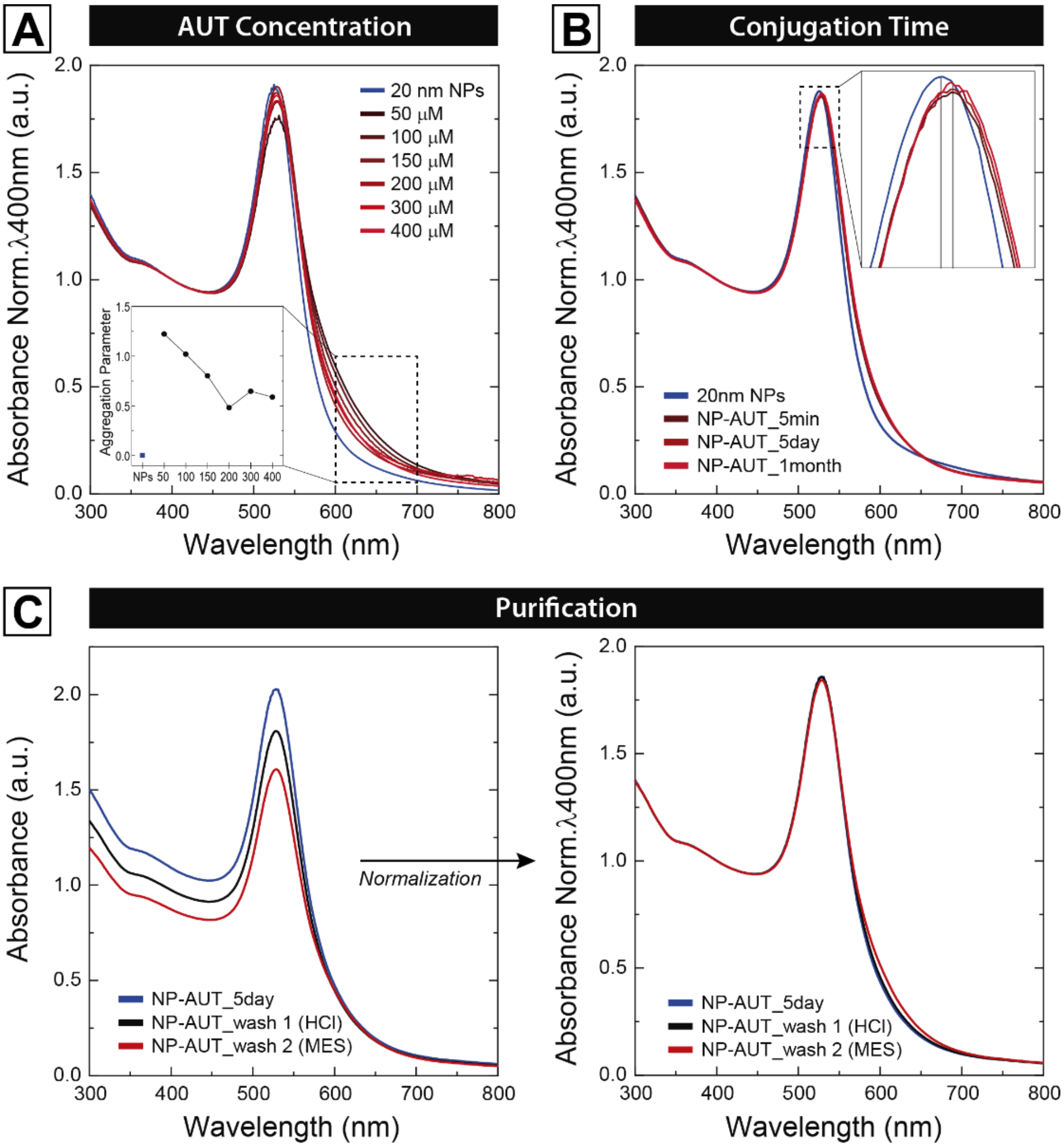

Figure S1. Study of the parameters affecting the conjugation of 20 nm Au NP to AUT. (A) AUT Concentration. Au NPs were conjugated to increasing concentrations of AUT (50-400µM) and characterized by UV-Vis spectroscopy after 24h. On the inset, the aggregation parameter was calculated based on the absorbance in the λ600-700 nm range related to the initial value. **(B)** Conjugation time. Au NPs were conjugated to AUT and characterized by UV-Vis spectroscopy after 5 min, 5 days or 1 month. **(C)** NP Purification. UV-Vis spectra after each purification step, normalized to λ400 nm (right) for better comparison.

Au NPs Conjugation to PEI.

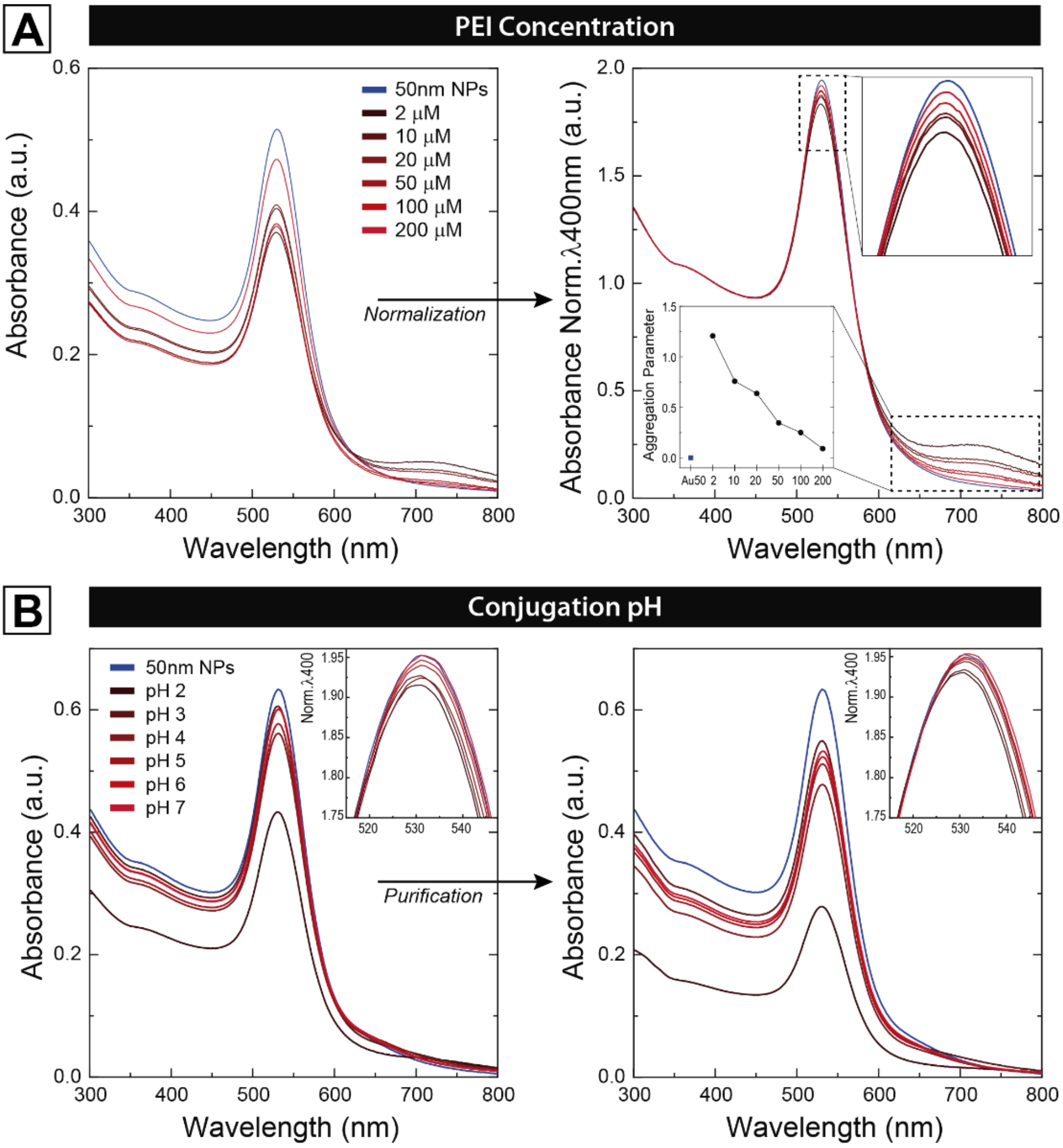

Figure S2. Study of the parameters affecting the conjugation of 50 nm Au NP to PEI. (A) PEI Concentration. Au NPs were conjugated to increasing concentrations of PEI (2-200µM) , characterized by UV-Vis spectroscopy after 24h, and normalized to λ400 nm (right) for better comparison. On the inset, the aggregation parameter was calculated based on the absorbance in the λ600-800 nm range related to the initial value. **(B)** Conjugation pH. Au NPs were conjugated to PEI at different pH values. After 24h, Au NPs were characterized by UV-Vis spectroscopy and after a purification step (right).

50 nm Au NPs were conjugated to a range of PEI concentrations from 2 µM to 200 µM to explore the optimal conditions to obtain a cationic conjugated NP that is highly stable in colloidal form. Results from the UV-Vis spectroscopy characterization are depicted in Figure S2A. A significant decrease in the absorbance intensity of the SPR peak can be observed as the concentration of PEI was decreased compared to the initial citrate-capped NP solution. It is also correlated with the

appearance of a “shoulder”, a localized absorbance increase, in the 600-800nm wavelength region, which is closely related to the aggregation state of the NPs in the form of “stable NP aggregates”. The normalization of absorbance allows better visualization of this phenomenon, and the calculation of the AP defines it quantitatively. An evident evolution towards less aggregation is observed with increasing concentrations of PEI, with a minimum AP value at 200 µM very close to the initial state. Thus, considering the results obtained, 200 µM was considered a good PEI concentration for NP conjugation since the decrease in absorbance is minimal and there are no signs of aggregation.

To reduce the risk of NP aggregation during the conjugation process, milder pH conditions were explored by conjugating 50nm Au NPs to 200 µM PEI at different pH values ranging from 2 to 7 (Figure S2B). Plots from the raw intensity absorbance (left) show a decrease in the SPR peak intensity that correlates with the decrease in pH, suggesting the aggregation of NPs at low pH. This can be seen when comparing normalized spectra at λ400nm (inset), revealing a slight decrease in the maximum absorbance of the SPR peak that indicates partial NP aggregation. NP aggregation increases as pH drop, evident by the loss of absorbance in the purified conjugates (right). Centrifugation brings NP under rather stressful conditions that might cause irreversible aggregation of the poorly stable colloids. The concentration of HCl needed to titrate the pH at the desired value. Consequently, the ionic strength caused by the coupled Cl^- concentration, significant changes from the most acidic point (700 mM) to the neutral point (34 mM). Therefore, at lower ionic concentrations, aggregation is less prone to occur and the efficiency of stable PEI-functionalized NP (NPs-PEI) increases.

Optimization of the Oligonucleotide:NP ratio.

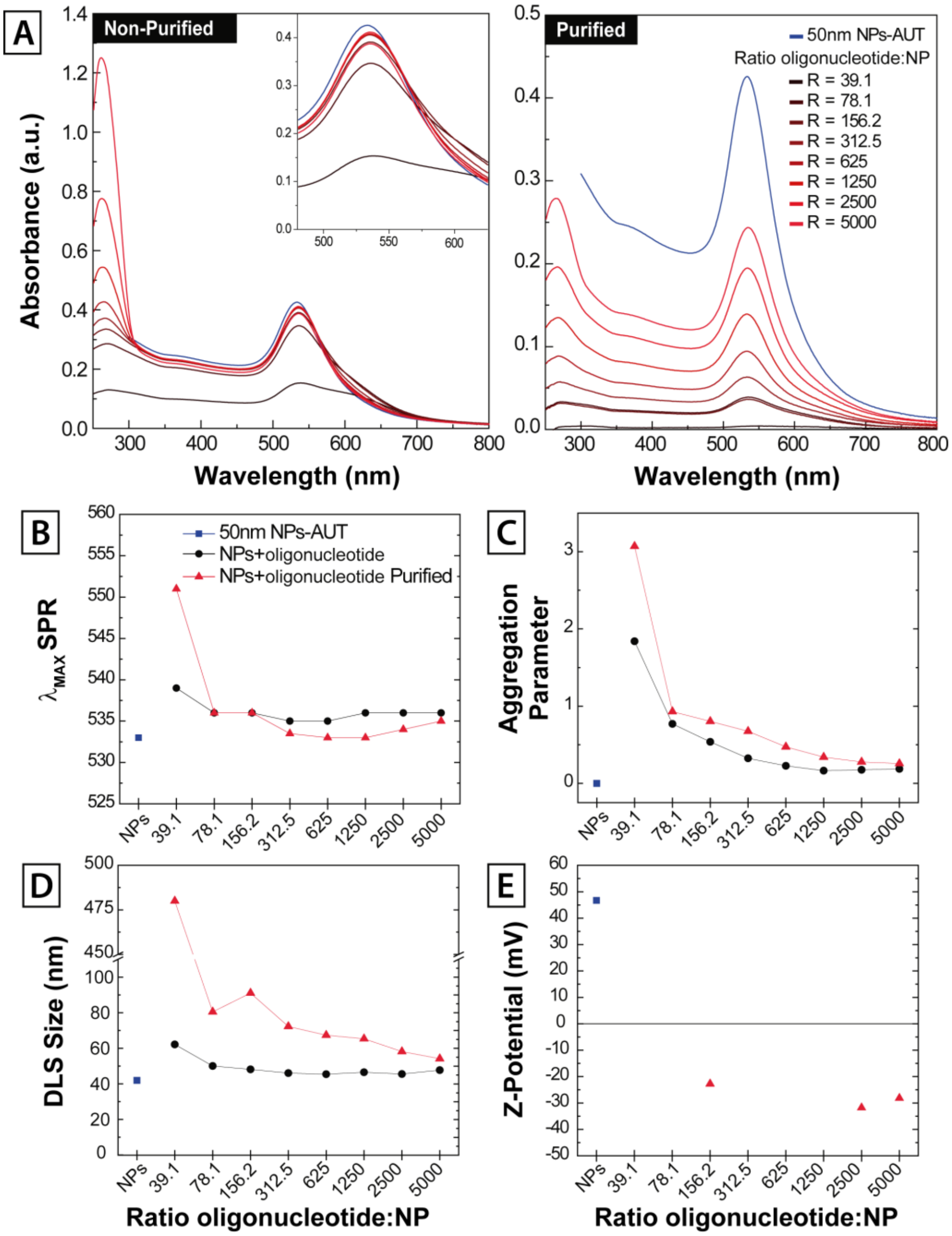

Figure S3. Loading of oligonucleotide to Au 50nm NPs-AUT. (A) UV-Vis spectra of Au NPs after 24h of incubation with increasing oligonucleotide:NP ratios, before and after NP purification. **(B)** SPR peak position at each ratio. **(C)** Calculated aggregation parameter based on the absorbance in the λ600-700 nm range of each ratio related to the initial value. **(D)** Experimental average DLS diameters for each ratio. **(E)** Zeta-Potential of the purified conjugates after 24h of incubation.

As a first approach to developing a conjugation protocol, 50 nm NPs-AUT were conjugated to increasing concentrations of oligonucleotide. This experiment aimed to determine an optimal ratio of oligonucleotide molecules to NP. The incubation was performed at 4 ºC to minimize the

risk of nucleic acid degradation and denaturalization and under mild stirring conditions to avoid NP sedimentation over time. The conjugation time was set to 24h to ensure surface saturation. Thus, the only changing parameter was the concentration of oligonucleotide from 0.41 to 53 µg/mL. The characterization of samples by UV-Vis (Figure S3A-C) and DLS (Figure S3D) suggest that the stability of the conjugates increased as the oligonucleotide:NP ratio increased. This spectrum is characterized by a broadening in the LSPR peak and a huge shift in the peak position. Remarkably, aggregation intensifies after NP's purification. As the ratio oligonucleotide:NP increase, there is a trend towards a more stable conjugate. The calculated aggregation parameter (Figure S3C) correlates well with these findings. It stabilises at the same ratio value as the ΔSPR and ΔDLS size, showing more aggregation in the purified samples. Likewise, Z-Potential measurements (Figure S3E) confirm the adsorption of oligonucleotide at the surface of the NPs, at any given ratio.

To set a good oligonucleotide:NP ratio for the standardization of a loading protocol, the main aspect to consider is the concentration of oligonucleotide that ensures that there is enough excess of molecules to conjugate to the NPs to avoid uncontrolled aggregation. Considering that i) the stability of NPs increases as the oligonucleotide:NP ratio increases until reaching a critical concentration in which the NPs remain stable, and ii) stability is assessed by UV-Vis spectroscopy and DLS, we study the critical point is where the ΔSPR and DLS size don't change compared to the following higher ratio, and the AP is low. Same experiments were performed for 5nm and 20nm cationic NPs (data not shown). Therefore, the optimal oligonucleotide:NP stablished for NP conjugation was **R=5 for 5nm-NP, R=50 for 20nm-NP and R=300 for 50nm-NP.** These ratios also correlate for oligonucleotide/nm^2 of NP surface.

Loading kinetics.

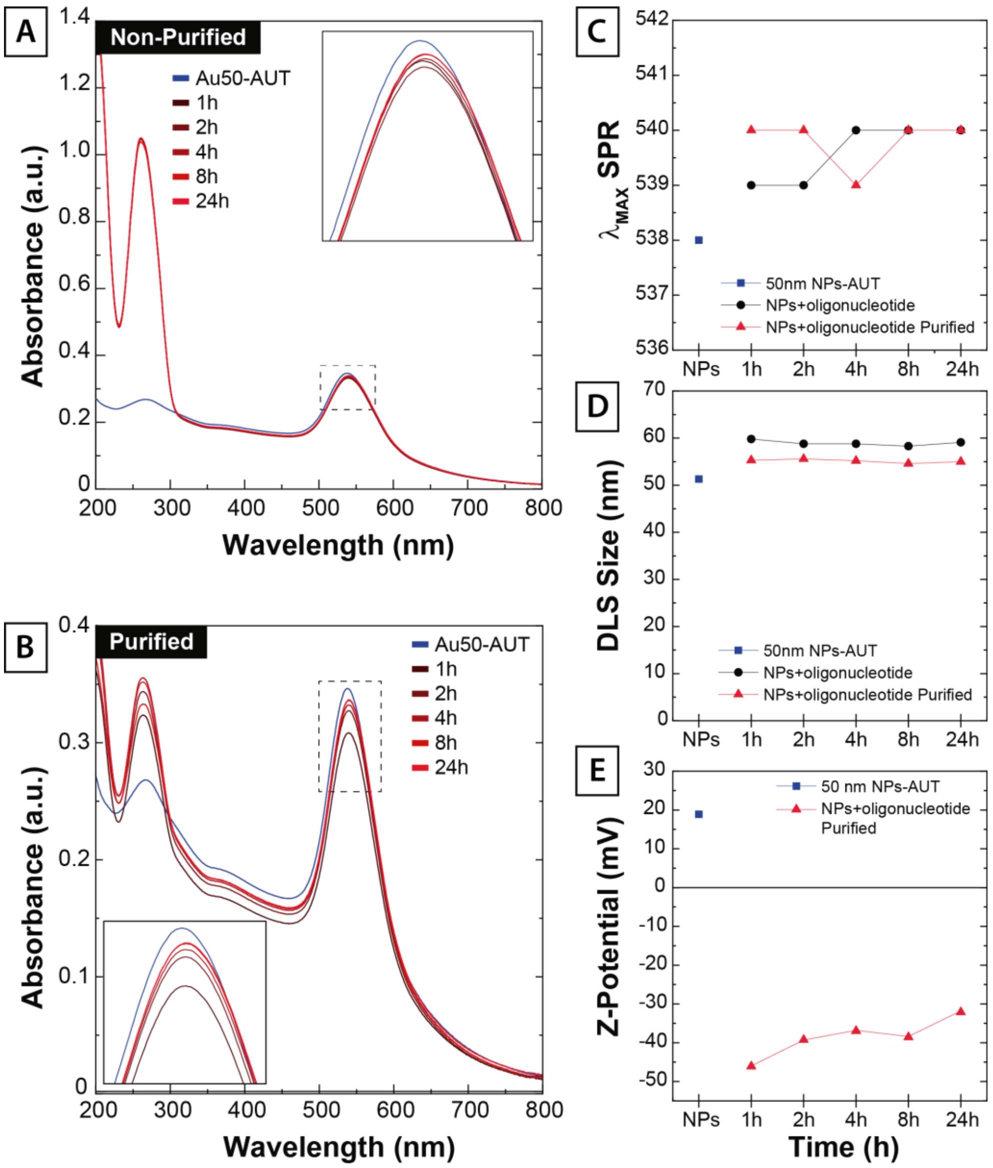

Figure S4. Loading kinetics of Au 50nm-AUT NPs to oligonucleotide. UV-Vis spectra of Au NPs loaded with oligonucleotide at each time point of incubation, before **(A)** and after NP purification **(B).** The presence of oligonucleotide in the solution is revealed by an appearance of a peak at λ260nm. **(C)** SPR peak position of Au NPs at time point. **(D)** Experimental average DLS diameters at each time point. **(E)** Zeta-Potential of the purified conjugates at each time point.

The results from the loading of NPs-AUT are displayed in Figure S4, where it can be observed how the interaction of oligonucleotide and cationic NPs is fast. This can be seen by evaluating changes in SPR peak position (Figure S4C), DLS size (Figure S4D) and surface charge measured by Z-Potential (Figure S4E) at the different time points studied.

A red-shift in the SPR peak position is observed and zoomed in the inset for a better visualization, which clearly indicates a loading of oligonucleotide on the NP's surface, as discussed in the

previous section. As plotted in Figure S4C, it corresponds to a Δ1-2 nm that stabilizes at 8h. Also, a high-intensity peak at λ260nm appears, corresponding to the presence of oligonucleotides in the solution. Conversely, after a purification step of the sample, changes in the measured UV-Vis spectra over time can be seen. At short times, the absorbance intensity is decreased but progressively increases over time, almost to the initial value. As explained, the instant shift of the peak position indicates that the interaction between NP-AUT and oligonucleotides is fast. Yet, the progression of the spectra after purification points out that the layer of loaded oligonucleotide might still be under an evolution process, probably a hardening of the corona. Similar results had been observed in previous studies regarding conjugating proteins to Au NPs.

Similarly, the DLS measured size increase is obvious from the first time point (Figure S4D). DLS size values did not change significantly over time. However, a constant slight decrease in size is observed after the purification of the sample due to the displacement of the dynamic equilibrium of the soft oligo-corona. Finally, clear evidence of the loading of oligonucleotides is the drop in the surface charge (Figure S4E). The measured Z-Potential abruptly changes from +19mV to highly negative values below -30mV. The presence of oligonucleotides on the surface of the NP-AUT, which is negatively charged at this pH value (~5), defined the charge of the overall construct.

Aiming to calculate the loading of oligonucleotide to cationic Au NPs, a quantification was performed by Nanodrop. For this, at the end of the incubation time (24h), supernatant from the purification step were collected and the oligonucleotide concentration was measured. Thus, the loaded oligonucleotide quantification was extrapolated from the free non-loaded oligonucleotide in the supernatant fraction. Nanodrop results after exposing cationic NPs at $3x10^{11}$ NP/mL to 19.15 ng/µl reveal a loading of 48% (9.3 ng/µl) for NPs-AUT and 45% (8.7 ng/µl) for NPs-PEI.

Stability of nanovectors.

The colloidal stability of the nanovectors was tested in different biological relevant media. This provides a better understanding of their physicochemical properties on transfection cell culture media, ultimately allowing a better understanding of the results when used as transfection vectors. Cationic functionalised 50 nm Au NPs, alone and loaded with oligonucleotides, were dispersed in phosphate buffer (PB) 10mM and Optimem and incubated for 24h. Although PB has the same pH as Optimem, the stability in both media was studied first to isolate the effect of the ionic strength of the media (as Optimem has a very high saline concentration) and also to avoid the potential interference that the presence of phenol red could cause.

The UV-Vis spectra from the characterisation of the samples are illustrated in Figure S5. Cationic NPs, both AUT and PEI-coated, aggregate when dispersed in PB or Optimem. The SPR peak critically broadens, and it shows a massive increase in the absorbance intensity in the 600-800nm region. Complementary characterisation of the samples by DLS and Z-Potential confirms these results, summarised in Table. The measured hydrodynamic size of the NP increases to values bigger than 400nm. The visual examination of the sample also corroborated this since aggregates precipitated fast to the bottom of the cuvette. The surface charge of NPs-AUT shifts to low negative values as a response to the pH increase to 7.4, whereas NPs-PEI remain in the positive range but with a value close to neutral. The loss of net surface charge, coupled to the high salinity of Optimem, led to NP aggregation due to the lack of electrostatic repulsion. Note that Z-Potential cannot be performed to NPs dispersed in Optimem due to its huge ionic strength, thus conductivity, of the media.

The stability of the nanovectors in physiologically buffered media significantly improved when cationic NPs had been previously loaded with oligonucleotide. For the AUT-coated nanovectors, although there is a drop in the surface charge of the nanovectors, SPR peaks show no signs of aggregation. A slight increase in the DLS size can also be seen after their dispersion in PB and Optimem as the nanovector loads oligonucleotide. The formation of an oligonucleotide corona on the surface of the cationic NPs provides steric repulsion to the conjugates and prevents their aggregation even in the most hostile conditions. However, as for the case of PEI-functionalized nanovectors, some destabilisation can be observed when dispersed in PB and Optimem. The absorbance of the UV-Vis spectra increases in the longer wavelengths, and DLS size also grows.

Note that, here, only 50 nm NP was used as a model to show nanovector stability in biological media. Further experiments performed with the rest of the NP sizes displayed similar results regarding the cationic coating and the presence of oligonucleotide corona.

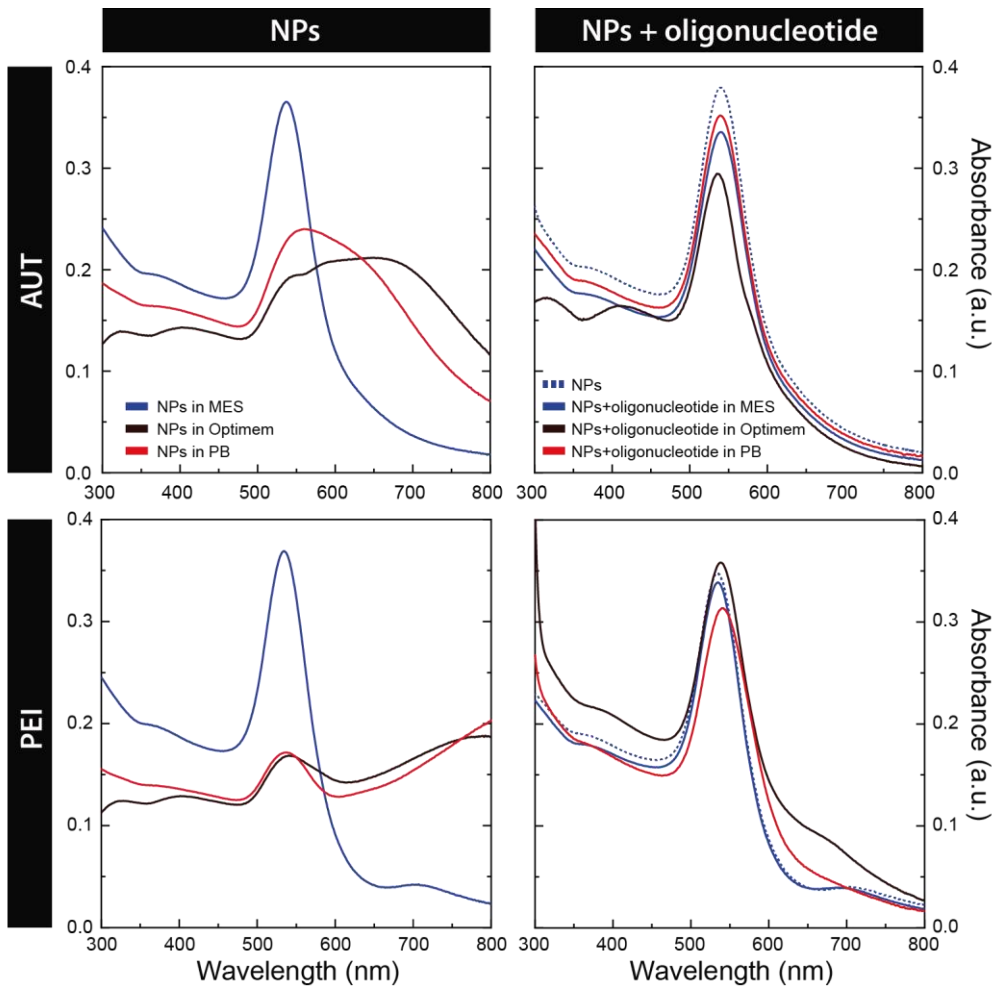

Figure S5. Stability in biological media. UV-Vis spectra of the cationic-coated Au NPs, alone or loaded with mRNA after 24h of incubation in Optimem and phosphate buffer (PB) 10 mM. MES buffer 10 mM was used as a reference stability control.

Table S1. Nanovector Stability Summary. Summary of sizes and surface charge of the cationic-coated Au NPs, alone or loaded with mRNA after 24h of incubation in Optimem and phosphate buffer 10 mM.

	NPs		NPs + Oligonucleotide	
	DLS	Z-Pot	DLS	Z-Pot
AUT				
in MES	69,6 ± 29,8	+31,2	78,1 ± 32,0	-28,6
in Optimem	437,1 ± 261,1	-	95,2 ± 42,0	-
in PB	491,5 ± 165,7	-11,1	86,2 ± 35,3	-3.4
PEI				
in MES	69,6 ± 28,4	+24,9	65,8 ± 25,7	+34,9
in Optimem	-	-	97,1 ± 43,0	-
in PB	433,3 ± 197,4	+9,7	103,7 ± 59,4	+3,9

Release.

Previous results suggest that the interaction between both entities is strong, based on electrostatic forces. However, once nanovectors have reached the cytoplasmic space, a critical point for the success and efficiency in protein expression is the release of the adsorbed mRNA from the cationic NP conjugates, mainly triggered by pH increase. To study the release of oligonucleotide from the nanovector construct over time, 50 nm Au NPs coated with AUT and PEI were first loaded with oligonucleotide. After 24h of incubation, nanovectors were dispersed in PB 10mM (pH 7.4) to a final relative volume of 10%. These experimental settings mimic the physiological conditions in transfection experiments regarding the pH of the culture media and NP dilution within it.

These results point out a slow release of oligonucleotide (thus probably mRNA) from the nanovector that would contribute to delayed delivery of the mRNA, which could extend the expression of the coded protein. Besides, a big fraction of the loaded oligonucleotide is still bound on the nanovector. In this regard, endocytosis of NP usually happens at short times, approximately within the first 4h, when the release rate is still rather low. This would ensure that most of the mRNA release on the transfection would happen inside the cell.

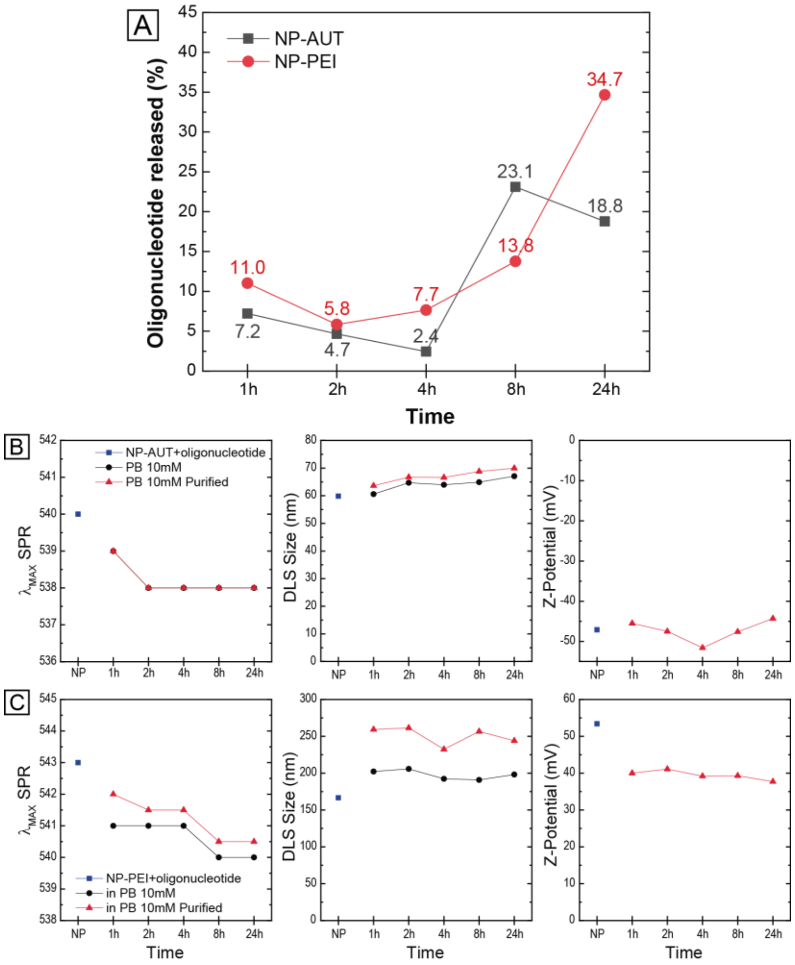

Figure S6. Oligonucleotide Release Kinetics. (A) Oligonucleotide released from the cationic nanoparticle over time, with respect to the total oligonucleotide loaded. Release kinetics were studied as a function of time, characterised by UV-Vis spectroscopy, DLS and Z-Potential. SPR peak position, measured average size and surface charge at each time point are plotted for AUT **(B)** and PEI **(C)** coated Au NPs.

As shown in Figure S6A, there is a sustained increasing release of oligonucleotide over time for both types of nanovectors. The release is represented as a percentage of the quantified oligonucleotide load. NPs-PEI shows a slightly higher rate of release, more remarkable at 24h. The detachment of oligonucleotide from the cationic NP is probably triggered by the pH increase, which lowers the net surface charge of the cationic coating. The loss of positive charge weakens the electrostatic interaction between the oligonucleotide and the NP, which displaces the dynamic equilibrium towards an oligonucleotide release in the dispersion media.

The characterisation of the nanovectors at different time points of exposure to PB 10 mM also revealed changes in their physicochemical properties. Their evolution was monitored by UV-Vis spectroscopy, DLS and Z-Potential, and the results are summarised in Figure S6B for the AUT-

coated NPs and in Figure S6C for the PEI-coated NPs. In both cases, the SPR peak position blue-shifts progressively up to Δ2nm, indicating a modification of the NP's surface that correlates well with a partial detachment of the oligonucleotide corona. On the other hand, an increase in the DLS size can be observed, which exacerbates after the purification process. This could be explained as a loss in the colloidal stability of the nanovectors during oligonucleotide release in the media of study. Finally, inconsistencies are found regarding the Z-Potential measurements. There are slight fluctuations in the surface charge of the NPs-AUT upon dispersion in PB 10 mM, but the net charge remains in the negative range. This agrees with the fact that only a fraction of oligonucleotides has detached from the nanovector. However, the NPs-PEI Z-Potential is consistently positive despite the loading of oligonucleotide, as previously discussed. There is a considerable drop from +53 mV to +40 mV since the overall surface charge of this construct corresponds to the PEI, which could be explained by a loss of charge due to the pH increase.

mRNA Concentration.

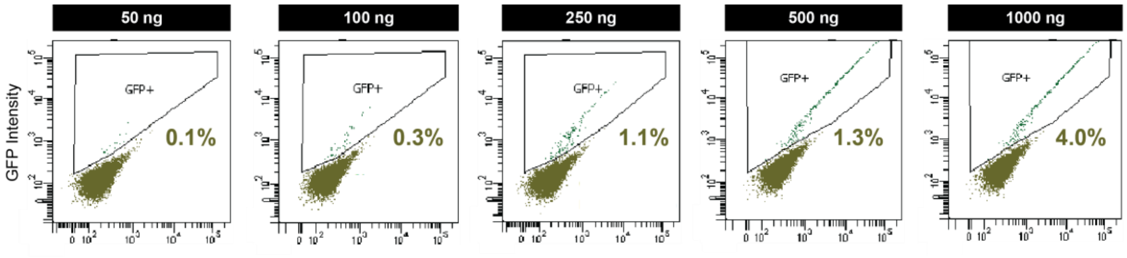

Figure S7. Optimization of *GFP* mRNA Transfection to HEK293 cells. GFP expression (% from the total cells population) at 24h is shown after transfection with 50-1000 ng mRNA. Nanovectors used for the transfection were 5 nm PEI-NP.

In order to optimize the transfection protocol, the transfection rate was studied as a function of the mRNA mass, which was increased from 50 to 1000 ng. As is can be observed in Figure S7, with 5 nm PEI-nanovectors the transfection efficiency increases from 0.1% to 4% as the mass of mRNA employed increases. In addition, these results revealed that despite the large amount of mRNA used, the ribosome is still not saturated, probably due to the slow release of the construct in the cytosol.

Confocal Imaging of mRNA-GFP Transfected HEK293 cells.

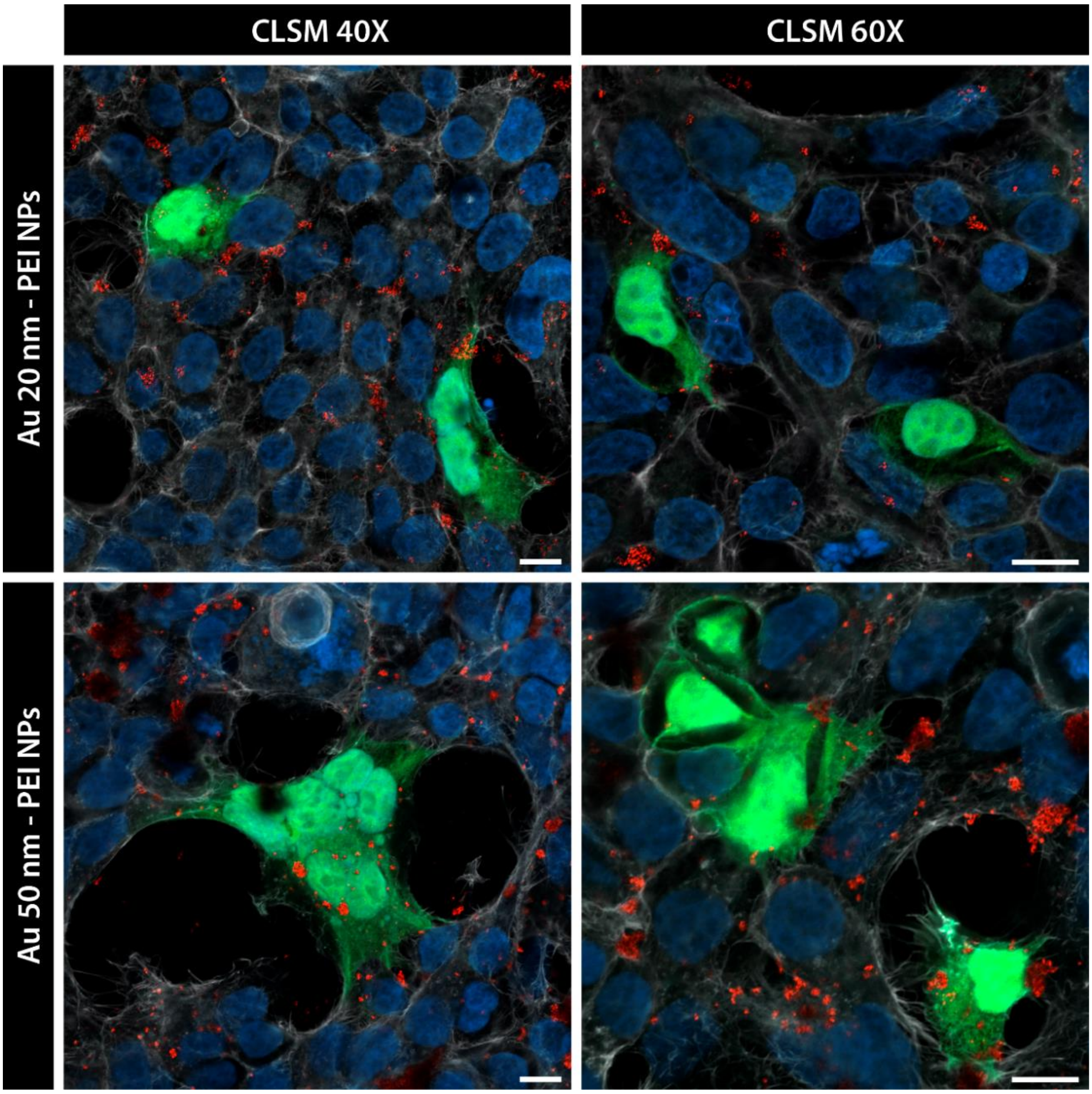

Figure S8. Confocal Imaging of mRNA-GFP Transfected HEK293 cells. GFP (green) expression is observed in transfected cells with 20 nm and 50 nm PEI-coated nanovectors (red). For cell structure, actin (white) and nuclei (blue) were stained. Scale Bar = 10 um.

The expression of GFP after the mRNA transfection on HEK293 cells was also visualised by Confocal Laser Scanning Microscopy (CLSM), which enabled to image of the Au NPs simultaneously by reflectance mode. In this case, 20 nm and 50 nm NPs-PEI nanovectors were used. After 24h, cells were fixed and stained to reveal the cellular structure, nuclei and actin. Representative images at 40X and 60X magnifications are shown in Figure S7. Au NPs, either 20 nm or 50 nm, can be found homogenously distributed throughout the sample but mainly in the intracellular space. Conversely, no significant higher NP uptake by GFP+ cells is observed compared to the non-expressing population.

On the other hand, magnification of the samples reveals that, despite the variability in GFP signal intensity between different cells, GFP expression is homogenously distributed within a single transfected cell. Differences in NP signal intensity between 20 nm and 50 nm NPs can be observed. Smaller NPs have lower intensity since they have very low or none light scattering contribution. Thus, they are only found in the intracellular space or in an aggregation state, generally located inside vesicles where NP concentration is high.

Transfection to different cell lines.

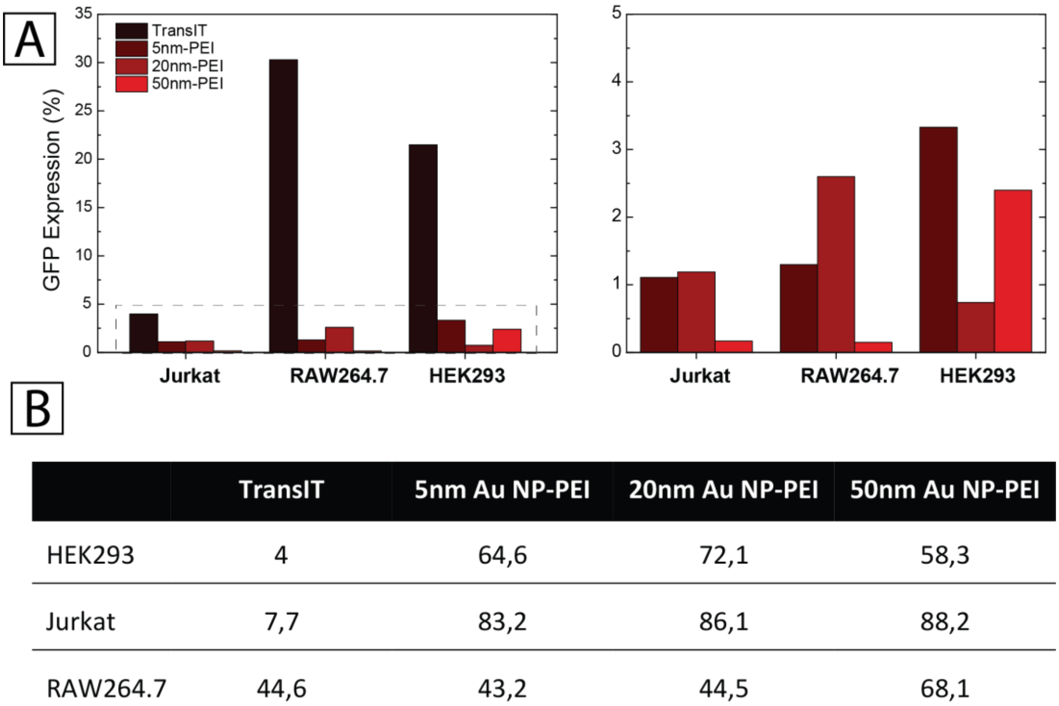

B

	TransIT	5nm Au NP-PEI	20nm Au NP-PEI	50nm Au NP-PEI
HEK293	4	64,6	72,1	58,3
Jurkat	7,7	83,2	86,1	88,2
RAW264.7	44,6	43,2	44,5	68,1

Figure S9. (A) *GFP* mRNA Transfection to Jurkat, RAW264.7 and HEK293 cells. GFP expression (% from the total cells population) at 24h is shown after mRNA transfection with 5, 20 and 50 nm PEI-derived nanovectors. **(B) Cell viability after transfection.** Values correspond to relative live cells at 24h after transfection with TransIT, and PEI-nanovectors of 5, 20, and 50 nm.

The expression of GFP at 24h after the mRNA transfection on Jurkat, RAW264.7 and HEK293 cell lines was analyzed by flow cytometry (Figure S9A). On previous experiments, HEK293 cells were used as an easily transfected cell line, as a model to test the full panel of the nanovector catalogue. Here, PEI-derived 5, 20 and 50 nm nanovectors loaded with *GFP* mRNA were used, since they showed the highest transfection efficiency. It is worth noting that both cell lines correspond to immune cells profile, T-cells and macrophages respectively, which pose a greater challenge for transfection. On the other hand, cell viability after 24h transfection (Figure S9B) is higher with nanovectors. Thus, these results allow to further prove the versatility of the nanovector in safely delivering mRNA to different cell types.

Materials and Methods

Chemicals

Gold(III) chloride trihydrate ($HAuCl_4 \cdot 3H_2O$), trisodium citrate ($Na_3C_6H_5O_7$), tannic acid ($C_{76}H_{52}O_{46}$), potassium carbonate (K_2CO_3), amino-undecanethiol (AUT), poly-ethyleneimine branched Mn2000 (PEI), 2-(N-morpholino)ethanesulfonic acid buffer solution (MES), sodium hydroxide (NaOH), hydrogen chloride (HCl), oligonucleotide model 600-800 bases (D1626), Sodium Phosphate Dibasic (Na_2HPO_4), Sodium phosphate monobasic (NaH_2PO_4), poly-L-lysine, Paraformaldehyde (PFA), Triton-X, Bovine Serum Albumin (BSA), Sodium Chloride (NaCl), and Calcium Chloride ($CaCl_2$) were purchased from Sigma-Aldrich. Dulbecco's Modified Eagle Medium (DMEM), Foetal Bovine Serum (FBS), Roswell Park Memorial Institute 1640 Medium (RPMI), 4-(2-hydroxyethyl)-1-piperazineethanesulfonic acid buffer (HEPES), Hoechst 3342 (H1399), Prolong antifade mounting medium (11559306), Optimem Medium, Pacific Blue-Annexin V, Propidium iodide (PI), accutase, and Prestoblue were purchased from Thermo Fisher. Phalloidin Alexa Fluor 647 (ab176759) was purchased from Abcam. Clean CAP eGFP mRNA (5 moU) was purchased from Tebu-Bio. TransIT®-LT1 Transfection Reagent was purchased from MirusBio. All chemicals were used as received without further purification. Distilled water passed through a Millipore system (ρ = 18.2 MΩ) was used in all experiments. All glassware was first rinsed with acetone and then with Millipore water before use.

Functionalization of Nanoparticles

Functionalization of Gold Nanoparticles with AUT. First parameter explored for a stable functionalization of Au NPs was the concentration of AUT. For this, 20nm Au NPs were concentrated 10-fold relative to the synthesis concentration by centrifugation (conditions were set according the Stokes law for each particle size). Next, AUT solutions with concentrations ranging between 50-400 µM were prepared in HCl 10 mM (pH<3). NPs (10% to final volume) were rapidly added into the AUT solution under vigorous stirring. After 1h, samples were characterized by UV-Vis. Note that at pH values above 3, NPs aggregate and precipitate upon dispersion in the AUT solution. The positive charges of the amine residues of AUT interact with the negatively charged hydroxyl residues of citrate and crosslink triggering NPs aggregation. The conjugation time was analyzed by monitoring the NPs by UV-Vis from 5 min to 1 month. Finally, the purification process of the AUT-coated NPs was studied. The conjugated NPs were precipitated by centrifugation twice, and resuspended to the initial volume, first with HCl 2 mM and then with MES 10 mM.

Functionalization of Gold Nanoparticles with PEI. The optimal PEI concentration and pH were studied for Au NPs PEI-coating. On the first case, 50nm Au NPs were concentrated 10-fold relative to the synthesis concentration by centrifugation. Next, PEI solutions with concentrations

ranging between 50-200 µM were prepared in HCl 34mM (pH~7). NPs (10% to final volume) were rapidly added into the PEI solution under vigorous stirring. After 1h, samples were characterized by UV-Vis. 10-fold concentrated 50nm Au NPs were conjugated to PEI (200 µM) at different pH conditions ranging from 2 to 7. NPs (10% to final volume) were rapidly added into the PEI solution under vigorous stirring. After 24h, samples were characterized by UV-Vis. The conjugated NPs were precipitated by centrifugation, resuspended to the initial volume with water and characterized again by UV-Vis.

Loading of Cationic Gold Nanoparticles with oligonucleotides

Optimization of the Oligonucleotide:NP ratio. 50 nm (at $3x10^{11}$ NP/mL) Au NPs coated with AUT were used, previously purified and dispersed in MES 10 mM. Non-coding oligonucleotide with a molecular weight (600-800 bases) similar to an average mRNA construct was used as a model. For oligonucleotide loading, first 900 µl of 2-fold serial dilutions in MES 10 mM were prepared, ranging from 53-0.41 µg/mL. Next, 100 µl of NPs were rapidly added onto the oligonucleotide and the mixture was gently homogenized. Thus, the final relative oligonucleotide:NP ratios ranged from [39-5000]. Samples were incubated for 24 h at 4ºC under stirring. Next day, samples were characterized by UV-Vis spectroscopy, DLS and Z-Pot before and after purification. For purification, NPs were precipitated by centrifugation, supernatant was discarded and pellets were resuspended in MES 10 mM to the initial volume.

Loading kinetics. 50 nm Au NPs coated with AUT were loaded with oligonucleotide. Briefly, 900µl of NPs dispersed in MES 10 mM were added onto 100 µl of oligonucleotide to a final ratio oligonucleotide:NP=300. Samples were kept at 4 ºC under stirring. At each time point, 1 mL of sample was taken for characterization. For purification, NPs were precipitated by centrifugation, supernatant was stored for oligonucleotide quantification and pellets were resuspended in MES 10 mM to the initial volume. Conjugates were analyzed by UV-Vis spectroscopy, DLS and additionally Z-Potential was measured after purification. The quantification of the oligonucleotide loaded on the NPs was extrapolated from the measurement of the supernatants at 24h by Nanodrop (Nanodrop 2000 Spectrophotometer, ThermoFisher).

Stability of nanovectors. To study the stability of nanovectors, 50 nm Au NPs coated with AUT and PEI, alone or loaded with oligonucleotide, were used. For this, NP solution was diluted 1:10 in the media of study and incubated for 24h at 4 ºC. Different biologically relevant media were tested: Optimem (pH 7.4) and Phosphate Buffer (PB) 10mM (pH 7.4). NPs dispersed in MES 10 mM (pH 5) were used as a control. NP stability was studied by UV-Vis and DLS. After 24 h samples were characterized. Au NPs were precipitated by centrifugation, the pellets were redispersed in the media of study and Z-Potential was measured.

In vitro Experiments

Cells culture. HEK293 and RAW264.7 cell culture was maintained in culture in 75 cm^2 tissue culture flask using DMEM supplemented with heat-inactivated foetal bovine serum (FBS) at 10% at 37 ºC and humidified 5% CO_2. Jurkat cell culture was maintained in suspension culture in 75 cm^2 tissue culture flask using RPMI supplemented with heat-inactivated foetal bovine serum (FBS) at 10% and 10 mM HEPES, at 37 ºC and humidified 5% CO_2.

Transfection Efficiency

Transfection of mRNA with nanovectors. To evaluate the transfection capacity of gold nanoparticles coated with PEI and AUT, cells were cultured in culture media with FBS 10% in 24-well plate. Cells were seeded at density: HEK293 at 50.000 cells/mL, RAW264.7 at 60.000 cells/mL, Jurkat at 30.000 cells/mL. The transfection was performed with 60-70 % confluence and final mRNA concentration of 1000 ng (otherwise specified). After 24h the incubation at 37ºC the DMEM medium was removed and replaced for 900 µL of Optimem medium. Next, specific colloidal ratios [mRNA:NP] were added (100 µL) for each nanoparticle size for 5 nm Au NPs [5:1], 20 nm [50:1] and 50 nm [300:1]. The next day 100µL of FBS were added to each well and left for 48h and 7 days after transfection process. The transfection and cell viability percentages were evaluated by confocal microscopy and flow cytometry.

Flow cytometry. The percentage of Green Fluorescence Protein (GFP) expression after transfection was analyzed with BD LSRFortessa™ Cell Analyzer. Forward and side-scatter areas (FSC-A, SSC-A) in a linear scale were used to gate HEK293, RAW264.7 and Jurkat population and GFP expression was detected by excitation through 480-500nm. To determinate the cell viability, HEK293 cells were stained with Pacific Blue - Annexin V/propidium iodide (PI) in accord with the manufacturer's recommendations. Briefly, cells were collected by cell detachment using accutase, scrapped or centrifuged, and washed with PBS. After centrifugation cells were resuspended in 100 µL of Annexin binding buffer (10mM HEPES, 140mM NaCl and 2.5mM $CaCl_2$). 5 µL of Annexin V and PI (1 mg /mL) were added and incubated at room temperature for 15 minutes. After the incubation period, additional 400 µL of the binding buffer was added. Acquisition was configured to stop after recording 10,000 events within the cell population.

Confocal Microscopy. Transfection of mRNA-GFP was performed on an 8-well glass bottom microslide. At 24h cells were fixed with 4% PFA. For immunohistochemistry, samples were first permeabilized with Triton-X 0.2% - BSA 1% for 10min. Samples were incubated with Phalloidin Alexa Fluor 647 (λ_{ex}650/ λ_{em}665) for 45min for actin staining. Nuclei were stained with Hoechst 3342 (λ_{ex}350/ λ_{em}461, dilution 1:10000) for 15min. Fading was controlled using the Prolong antifade mounting medium. Samples were observed on the Thunder Wide-Field Fluorescence Microscope (Leica) and on the Confocal Laser Scanning Microscope (Zeiss LSM980 with

Airyscan 2) in order to image the GFP (λ_{ex}488/ λ_{em}507) expression and NP distribution by light scattering on transfected HEK293 cells.
